# Multi-criteria protein structure comparison and structural similarities analysis using *pyMCPSC*

**DOI:** 10.1371/journal.pone.0204587

**Published:** 2018-10-17

**Authors:** Anuj Sharma, Elias S. Manolakos

**Affiliations:** 1 Department of Informatics and Telecommunications, National and Kapodistrian University of Athens, Athens, Greece; 2 Northeastern University, Boston, Massachusetts, United States of America; UMR-S1134, INSERM, Université Paris Diderot, INTS, FRANCE

## Abstract

Protein Structure Comparison (PSC) is a well developed field of computational proteomics with active interest from the research community, since it is widely used in structural biology and drug discovery. With new PSC methods continuously emerging and no clear method of choice, *Multi-Criteria Protein Structure Comparison* (MCPSC) is commonly employed to combine methods and generate consensus structural similarity scores. We present *pyMCPSC,* a Python based utility we developed to allow users to perform MCPSC efficiently, by exploiting the parallelism afforded by the multi-core CPUs of today’s desktop computers. We show how *pyMCPSC* facilitates the analysis of similarities in protein domain datasets and how it can be extended to incorporate new PSC methods as they are becoming available. We exemplify the power of *pyMCPSC* using a case study based on the Proteus_300 dataset. Results generated using *pyMCPSC* show that MCPSC scores form a reliable basis for identifying the true classification of a domain, as evidenced both by the ROC analysis as well as the Nearest-Neighbor analysis. Structure similarity based “Phylogenetic Trees” representation generated by *pyMCPSC* provide insight into functional grouping within the dataset of domains. Furthermore, scatter plots generated by *pyMCPSC* show the existence of strong correlation between protein domains belonging to SCOP Class C and loose correlation between those of SCOP Class D. Such analyses and corresponding visualizations help users quickly gain insights about their datasets. The source code of *pyMCPSC* is available under the GPLv3.0 license through a GitHub repository (https://github.com/xulesc/pymcpsc).

## Introduction

Protein Structure Comparison (PSC) allows the transfer of knowledge about known proteins to a novel protein. Novel protein structures are routinely compared against databases of known proteins to establish functional similarities using “guilt by association” [1]. Conservation of proteins is known to be much higher at the structure than at the sequence level, therefore structural similarity is essential in assigning functional annotations to proteins [2]. Function assignment is typically achieved by developing a template of the functional residues of the proteins and then aligning the template with complete known structures [3]. Structural comparison approaches are also increasingly employed in drug repositioning [4]. PSC methods are used to identify proteins with similar binding sites all of which then become potential targets for the same ligand [5, 6]. All these important applications require the structure of one or more proteins (queries) to be compared against a large number of known protein structures (one-to-all or many-to-many type comparison) to identify protein pairs with high structural similarity.

Performing large-scale PSC experiments (with thousands of protein structures) is time consuming due to three factors: a) the time complexity of the individual pairwise problem, b) the trend towards providing consensus results using multiple methods and c) the exponential growth of structural databases. The problem of aligning two protein structures is known to be NP-hard [7]. Over the years, many heuristic methods have been proposed for pairwise PSC. They vary in terms of algorithms and similarity metrics used, yielding different but biologically relevant results. Thus, no single method is currently considered superior for PSC [8]. So, it is common to generate consensus results by combining different methods, a trend known as *Multi-criteria Protein Structure Comparison (MCPSC)* [9]. With the advances in high-throughput technologies, the number of known protein structures is growing very rapidly [10]. This is reflected in the size of the Protein Data Bank (PDB) [11] is increasing exponentially (Fig B in S1 File). Given the great importance of PSC in diverse fields, there is a need for efficient MCPSC techniques and software to identify structurally similar protein pairs within a large dataset.

To this end, a useful cluster computing shared resource available to the community is the ProCKSI server [12]. Given a dataset of protein domains, it supports *all-to-all* MCPSC experiments, returning to the user individual PSC method scores as well as a consensus average score. While ProCKSI is an one-stop resource, it is limited in the size of the data that a user is allowed to submit (upto 250 protein domains). Moreover, the users cannot add new PSC or MCPSC methods of their choice. In general, distributed solutions, implemented using shared resources, suffer from limitations such as extensibility and maintainability.

In order to exploit modern processor architectures (CPUs) we have ported in [13] a popular PSC method (TM-align [14]) to an experimental many-cores CPU architecture, namely Intel’s Single-chip Cloud Computer (SCC) [15], a processor having 48 cores interconnected via a mesh-type Network-on-Chip. We extended this work to support efficient MCPSC on the SCC processor in [16]. However, to the best of our knowledge, there is currently no software utility available to the community for flexible and parallel MCPSC that can exploit the ubiquitous multi-core CPUs of today’s PCs and can be extended with new PSC methods to provide systematic MCPSC similarity analysis of large protein datasets.

In this work, we introduce such a utility, called *pyMCPSC,* which we have created using the popular Python programming language [17] and make available to the community. *pyMCPSC* generates pairwise structure comparison and consensus scores using multiple PSC and MCPSC methods. In addition, the resulting similarity scores are used to generate multiple insightful visualizations that can help a) compare and contrast the structure comparison methods, and b) assess structural relationships in the analyzed dataset. Such comprehensive analysis allows researchers to gain quick visual insights about structural similarities existing in their protein datasets, simply by exploiting the power of multi-core CPUs of their computers.

*pyMCPSC* allows pairwise structure comparison tasks to be distributed over the multiple cores of the CPU and provides a simple Command Line Interface (CLI) for setting up and running all-to-all MCPSC experiments in a standard PC. Our utility wraps available executable PSC method binaries with a user specified class, thus making it easy to incorporate new PSC methods in MCPSC analysis while hiding the details of parallel job distribution from the user. As distributed today, *pyMCPSC* contains wrappers for the executable binaries of five well known PSC methods: *CE* [18], *TM-align* [14], *FAST* [19], *GRALIGN* [20] and *USM* [21]. These implementations can also serve as examples of how to quickly extend the utility with new PSC methods as soon as their binaries become available to the community. In addition, *pyMCPSC* generates consensus (MCPSC) scores using multiple (five) alternative schemes. Finally, *pyMCPSC* uses the computed similarity scores (PSC and MCPSC) to generate several insightful visualizations.

As a use case, we employed *pyMCPSC* to analyze the Proteus_300 dataset [22] and compare the performance of the PSC methods and consensus schemes (MCPSC) currently supported. The dataset consists of 300 unique protein domains. We were able to match *N* = 270 of them to SCOP [23] classifications using Astral v1.75 [24]. A total of *P* = *N*^2^ − *N* domain pairs were then generated. We used this dataset because it is of a reasonable size and has also been used in previous PSC work [20] to compare PSC methods on speed and classification accuracy. We show that the generated consensus scores achieve a very high Area Under the Curve (AUC) and domain auto-classification accuracy (using Nearest-Neighbor classification) matching or exceeding that of the best component PSC method which in practice is not known for any given dataset. Our analysis shows that MCPSC methods provide consistent performance for structure-based protein classification. Moreover, scatter plots, heatmaps and “Phylogenetic Trees” generated by *pyMCPSC* in structural space reveal novel information about the presence of strongly associated domains within the dataset. Moreover, to demonstrate the capabilities of the utility to handle very large datasets we have processed the SCOPCATH dataset [25]. The results of the analysis are summarized in S2 File.

## Methods

### Design and implementation

**Fig 1:**
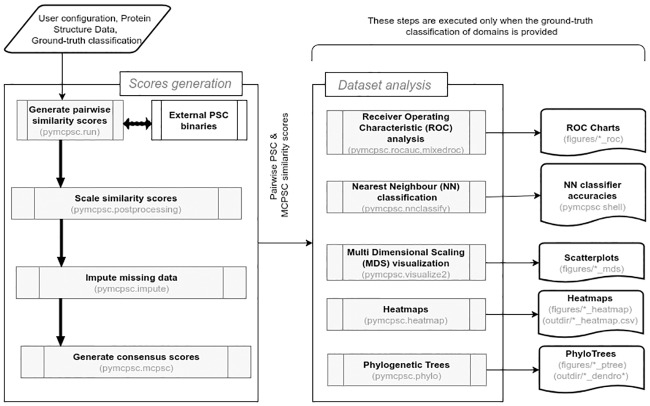
Schematic overview of the architecture of *pyMCPSC*. *pyMCPSC* is organized into several modules, each one implementing a specific functionality. The main entry point of the utility drives the sequence of activities shown. Similarity scores are generated for all protein pairs using the executable binaries of the included PSC methods. Subsequently the scores are scaled, missing data (similarity scores) are imputed and consensus MCPSC scores are calculated for all domain pairs. If the user has supplied ground-truth domain classification information, then comparative analysis results are also generated based on the similarity scores. The modules where the respective functionalities are implemented are specified in parenthesis.

As a software architecture, *pyMCPSC* is organized into several modules called in sequence by the main entry point. An overview of the processing sequence is shown in Fig 1. The modules are functionally independent and the interface between them is via files. Each module receives a set of parameters, including the files used to read data and write the output results. In a typical scenario, the user sets up an experiment, using command line parameters for supplying information such as the location of protein domain structures data and ground-truth classification (if available). The ground-truth data required by *pyMCPSC* to perform the analysis steps is the SCOP/CATH [26] classification of the domains in the dataset being analysed. The information is expected to be provided to the utility in a specific format. *pyMCPSC* first generates pairwise similarity scores for all domain pairs, using the supplied PSC methods and the implemented MCPSC methods, and then generates results to facilitate structure based comparison and analysis.

### Consensus scores calculation

Given a set of protein domains, *pyMCPSC* generates similarity scores using the supported PSC methods, for all protein pairs (all-to-all) that can be formed using the dataset. One-to-many, many-to-many and all-to-all PSC jobs with one or more PSC methods can be distributed in multiple ways depending on the unit of work sent to the processing elements [27]. If there are *P* pairwise comparisons to be made and *M* PSC methods to be used, the total number of fine grained pairwise PSC jobs is *P*×*M*. By default *pyMCPSC* creates a list of pairwise comparisons (*P*) corresponding to the all-to-all setup (all pairs of domains in the specified dataset). Pairwise similarity scores are then generated by calling third party external binaries for each of the supported PSC methods. The pairwise PSC processing is distributed over *p* threads (a configurable parameter), equal to the number of cores in the processor. The user may include specific pairs of interest in the ground-truth data (one-to-many or many-to-many setups) to limit the pairwise comparison results used in consensus calculations and performance analysis. Once all the pairwise similarity scores have been generated, the MCPSC consensus scores are also computed for the domain pairs. The consensus scores calculation involves several steps, as indicated in Fig 1.

### Data imputation

A “local average fill” scheme is used to compensate for potentially missing data for each PSC method. Missing PSC score for pairs of domains can be a result of PSC method executable or PDB file errors and can be problematic for classification/clustering analysis that rely on these values. Assuming that pairwise PSC scores were successfully generated for *s* domain pairs (out of the total *P* pairs in a dataset), the number of missing pairwise scores is *P* − *s*, with the value of *s* being different for different PSC methods. To impute the missing data for each PSC method, the following steps are repeated for all domain pairs (*d*_*i*_, *d*_*j*_) with a missing score:

- find the set of PSC scores where *d*_*i*_ is the first domain in the pair
- find the set of PSC scores where *d*_*j*_ is the second domain in the pair
- merge the two sets and use the mean value of scores in the set union as the PSC score for that domain pair
- if the two aforementioned sets are empty then use the global average of scores for that PSC method to supply the missing score’s value.

#### PSC scores

**Base PSC scores calculation** Pairwise scores for all PSC methods are first converted to dissimilarities (with value higher when domains in the pair are more different).

**PSC scores scaling** A Logistic Sigmoid scheme is used to scale scores to ensure equal contribution of PSC methods towards the consensus MCPSC scores calculation. Given the base dissimilarity score (*X*) for a PSC method, its scaled version (*S*) is obtained using Eq (1) below, where *μ* and *σ* are the mean and standard deviation respectively over all scores *X* for that method. Effectively, the dissimilarity scores are first autoscaled (to make the different PSC method scores comparable) and then the logistic sigmoid is applied. As a result, at the end we obtain similarity scores (*S*) in the range 0 to 1.

$$\begin{array}{c} S=1-\frac{1}{1+e^{-\frac{X-\mu }{\sigma }}} \end{array}$$ (1)

#### MCPSC consensus scores calculation

*pyMCPSC* produces five alternative MCPSC consensus scores as discussed below:

- **M1**—It is the Generalized Mean of the available PSC scores and is computed as shown in Eq (2) below, where *m* is the number of non-null PSC method scores available for a given domains pair. In the current implementation *q* = 1, hence *M*1 is essentially the average of the available PSC scores for the pair.
  $$\begin{array}{c} M1={\left(\frac{1}{m}\sum_{i=1}^{m}{S_{i}}^{q}\right)}^{\frac{1}{q}} \end{array}$$ (2)
- **M2**—It is a weighted average of the PSC scores of the different methods. For each domain pair we weight the available PSC method scores by the percentage of pairs successfully processed by each PSC method in the whole dataset (coverage based weighting).
- **M3**—Similar to **M2**, but here we also allow domain expert knowledge to play a role in the method’s relative weighting e.g. we lower USM method’s weight to one half since it is a domain agnostic method (domain expert knowledge based weighting).
- **M4**—For each domain pair, we weight each PSC method by the mean RMS distance of its scores from those of the other PSC method scores, as shown in Eqs (3), (4) and (5) below, where $S_{k}^{i}$ is the scaled PSC score for the *k*^*th*^ domain pair (*k* = 1, 2, …, *P*) and the *i*^*th*^ PSC method (*i* = 1, 2…, …*M*). If scores $S_{k}^{i}$ or $S_{k}^{j}$ are missing, the corresponding *k*^*th*^ term is excluded from the summation in (3) (divergence driven weighting).
  $$\begin{array}{c} RMSD_{ij}=\sqrt{\frac{1}{P}\sum_{k=1}^{P}{\left(S_{k}^{i}-S_{k}^{j}\right)}^{2}} \end{array}$$ (3)
  $$\begin{array}{c} r_{i}=\frac{1}{M}\sum_{j=1}^{M}RMSD_{ij},\;i,j\in \left\{1,2,\ldots ,M\right\} \end{array}$$ (4)
  $$\begin{array}{c} w_{i}=\frac{r_{i}}{max(r_{i})},i\in \left\{1,2,\ldots ,M\right\} \end{array}$$ (5)
- **M5**—For each domain pair, we weight the PSC methods by user supplied relative weights. For example, in the experiment with the Proteus_300 dataset [22] discussed in the Results section, the PSC method weights were learned by applying a logistic regression method where only 10% of the dataset was used for training to extract the relative method weights (see details in Section B.2 in S1 File).

For a domain pair with *m* available PSC method scores, where in general *m* < = *M* (*M* = 5 currently), the *m* weights are first normalized to sum up to one and the consensus score (for schemes **M2**-**M5**) is then calculated as the weighted average of the available *m* scores. MCPSC schemes **M1**-**M4** leverage different properties of their component PSC methods, while weighting them in different ways to generate a consensus score. Finally, a *median* MCPSC score per domain pair is generated using the **M1**-**M5** scores. As *pyMCPSC* sources are made available, it is also entirely possible for the user of the utility to experiment with new consensus score generation schemes.

### Comparative evaluation of different methods

The generated pairwise domain similarity scores (PSC and MCPSC) are written to a file (*processed.imputed.mcpsc.csv*) in the user defined output directory. If ground truth information i.e. true classification (such as SCOP and CATH) is available for each protein domain, *pyMCPSC* also performs the following data analysis steps (see Fig 1):

1. Generates Receiver Operating Characteristics (ROC) curves and computes the corresponding Area Under the Curve (AUC) values for each method.
2. Performs Nearest-Neighbor auto-classification of protein domains, at any specified level of SCOP hierarchy, using PSC/MCPSC score-based distance matrices.
3. Embeds the protein domains in a 2-dimensional space for visualization using Multi-dimensional Scaling (MDS) and generates scatter plots. Parallel computation is used to utilize all cores of the processor.
4. Generates Heatmaps, at Domain and Fold SCOP hierarchy levels, using PSC/MCPSC score-based distance matrices.
5. Generates “Phylogenetic Trees” of protein Domains, using PSC/MCPSC score-based distance matrices.

### Dependencies

*pyMCPSC* relies on extensively used scientific packages such as: Pandas [28], Scikit [29], Numpy [30], Seaborn [31], dendropy [32], Ete3 [33] and Matplotlib [34]. Binaries for the five default PSC methods are pre-packaged in *pyMCPSC,* however currently they are available only on machines running 64-Bit Linux O/S (limiting factor is GRALIGN). However, a docker container of *pyMCPSC* can be built and run on any operating system. Details on how to install the dependencies and *pyMCPSC* software are provided in Section A.2 in S1 File. *pyMCPSC* has been tested on Python 2 (version 2.7) and Python 3 (version 3.5). Further, build and installation instructions, application programmer interface (API) and documentation can be built using the Sphinx documentation system included in *pyMCPSC.*

## Results and discussion

We will demonstrate the use of *pyMCPSC* using protein pairs obtained from the Proteus dataset [22]. PSC scores were obtained for these pairs and analyzed as discussed in the paper. The number of pairwise PSC jobs processed per PSC method is actually one half of this value because of the symmetry of the PSC scores matrix, however the post processing and performance calculations are performed with the full matrix. The PDB files, the ground-truth SCOP classification and the pairwise domain list as well as the experimental setup are included in the *test* folder of the downloadable sources. *pyMCPSC* generates performance results for three sets of domain pairs, defined as follows:

- Original Dataset: It includes the similarity scores for the domain pairs defined in the original dataset, but with missing values. The number of missing values may vary depending on the PSC method as explained above.
- Common Subset: It consists of the subset of domain pairs taken from the Original dataset for which scores have been generated by all PSC methods. In the case of the Proteus dataset, this corresponds to 27312 domain pairs, which is less than half of the total number of pairs processed.
- Imputed Dataset: It consists of the Original dataset with the missing scores filled using data imputation The total number of domain pairs for the Proteus dataset is *P* = 72630.

### Performing MCPSC on a multi-core processor

Using *pyMCPSC* we generated pairwise similarity scores (all-to-all) based on the 5 PSC methods and the 5 MCPSC schemes (M1—M5) included in the utility by default, as well as the pairwise median MCPSC scheme. Experiments were carried out using multi-threaded processing on an Intel Core i7- 5960X “Haswel” 8-Core (16 Threads) CPU running at 3.0 GHz with 32 GB of RAM and an SSD running Linux. The Core i7 CPU features highly optimized out-of-order execution and HT (Hyper Threading), Intel’s flavor of Simultaneous Multi-Threading (SMT).

Details of setting up an experiment with *pyMCPSC* and the supported arguments can be found in Section A.4 in S1 File. The number of domain pairs for which scores were successfully generated varies among the PSC methods (Table 1), with GRALIGN and FAST having the lowest coverage. This is attributed to differences between the build and runtime environments, the thresholds built into the PSC method programs and errors in the structure files downloaded from the PDB. A speedup factor of 9.13 is achieved for end-to-end processing of the Proteus 300 dataset using *pyMCPSC* when *p* = 16 threads are used (Table 2).

**Table 1:** PSC methods coverage for the Proteus dataset.

PSC Method	# Domain pairs processed	Coverage
CE [18]	64964	89%
FAST [19]	39604	55%
GRALIGN [20]	56406	78%
TM-align [14]	72630	100%
USM [21]	72630	100%

**Table 2:** Time (in seconds) and Speedup (S) for end-to-end all-to-all analysis of the Proteus_300 dataset using *pyMCPSC* on a multi-core PC with Intel i7 CPU having 8 cores (16 threads), 32 GB RAM, running at 3.0 GHz, under Ubuntu 14.04 Linux. GRALIGN already uses all the CPU cores by default.

	1 Thread	4 Threads		8 Threads		12 Threads		16 Threads	
	Time	Time	S	Time	S	Time	S	Time	S
**Pairwise scores generation**									
GRALIGN	86	86	1.00	86	1.00	86	1.00	86	1.00
USM	139	46	3.02	20	6.95	17	8.18	15	9.27
FAST	4100	1035	3.96	412	9.95	329	12.46	313	13.10
TM-align	3601	1032	3.49	423	8.51	333	10.81	299	12.04
CE	16776	4022	4.17	1858	9.03	1420	11.81	1213	13.83
Consensus scores	28	28	1.00	28	1.00	28	1.00	28	1.00
**Block level**									
*Scores Generation*	24730	6249	3.96	2827	8.75	2213	11.17	1954	12.66
*Dataset Analysis*	843	849	0.99	844	1.00	846	1.00	847	1.00
**End to End**									
End to End	25573	7098	3.60	3671	6.97	3059	8.36	2801	9.13

Table 1 provides the number and percentage of pairs (coverage) successfully processed by each PSC method. Table 2 shows the time *pyMCPSC* needs to process the pairwise PSC tasks for the Proteus_300 dataset when using an increasing number of threads (from 1 to 16). GRALIGN is not run in parallel by *pyMCPSC* because its binary is already optimized to use all the available cores of the CPU. Entries in the table correspond to the blocks shown in Fig 1 of the manuscript. The table shows the time taken by the five PSC methods and the consensus scores calculation (Scale similarity scores, Impute missing data, Generate consensus scores). In addition to the end-to-end computation time we also provide in Table 2 the total times for the Scores Generation and the Dataset Analysis blocks (as defined in Fig 1). We believe that the superlinear speedup observed in parallel pairwise PSC processing is due to PDB structure data caching which allows multi-threaded runs reuse the cached files.

**Fig 2:**
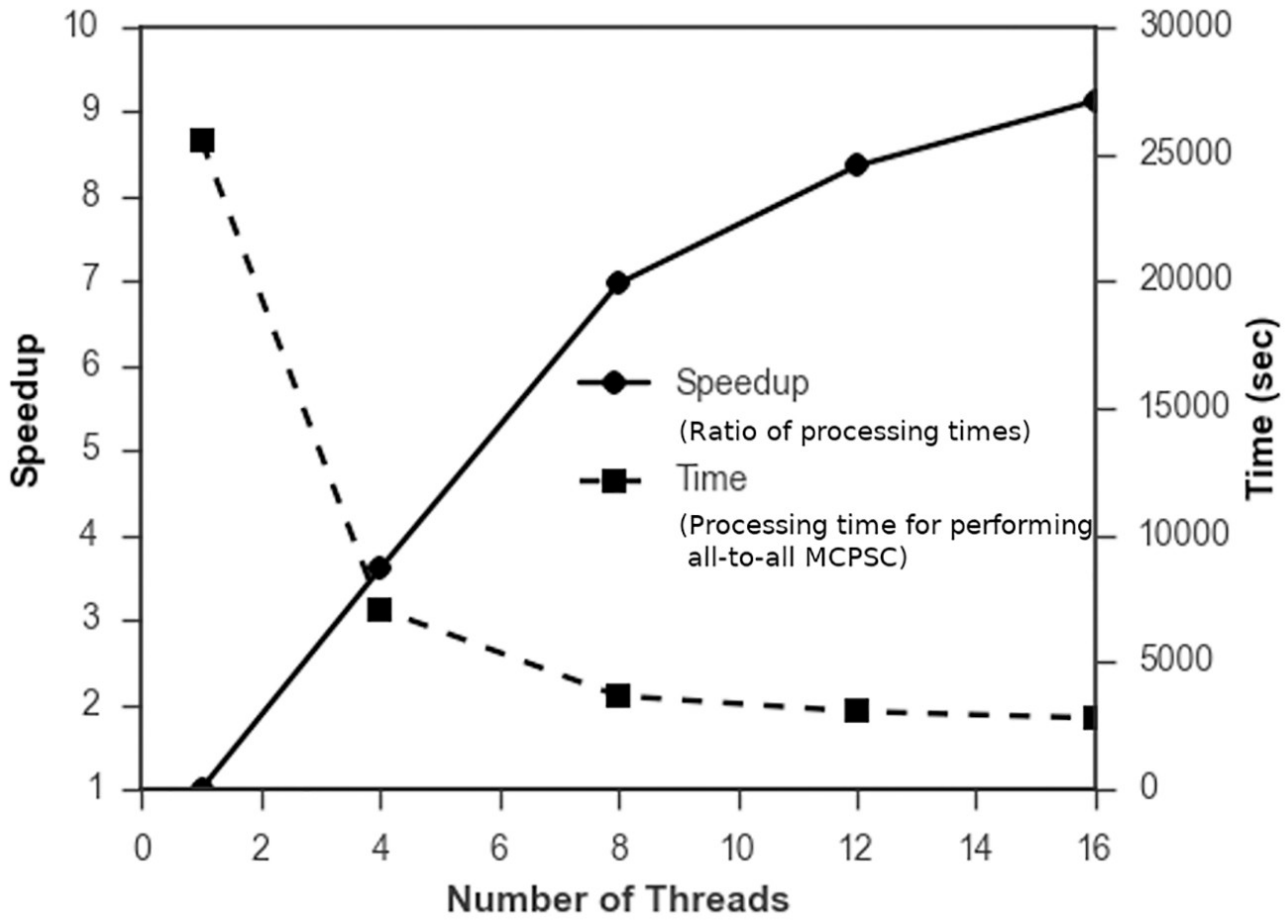
Speedup factor and total processing time for performing all-to-all MCPSC with increasing number of threads on a Intel Core i7 multicore CPU using the Proteus 300 dataset.

In Fig 2 we show the speedup factor achieved and the total processing time as the number of threads increases from 1 to 16. Nearly linear speedup is observed till the number of threads reaches the number of available cores of the CPU (8). The speedup continues to grow with the number of cores even beyond that point, albeit at a slower rate. This analysis suggests that the emerging many-core processors with more than 16 cores could also be exploited by *pyMCPSC* to analyze very large datasets.

### *pyMCPSC* generates quality consensus scores

Receiver Operating Characteristics (ROC) analysis can be used to compare the classification performance of MCPSC with that of the component PSC methods. *pyMCPSC* uses ROCs and corresponding Area Under the Curve (AUC) values for performance benchmarking if ground truth data is available.

The following procedure is used to create the ROC curves: a) Vary a similarity threshold from 1 down to 0, moving from maximum to minimum similarity; b) For each threshold value record the number of True Positives (TP), False Positives (FP), False Negatives (FN) and True Negatives (TN). In this context, TPs (FPs) are domain pairs with similarity scores greater than the set threshold in which the two domains in the pair have the same (different) classification at the SCOP hierarchy [35] level considered respectively. Similarly, FNs (TNs) are domain pairs with similarity score less than the threshold having same (different) domain classifications respectively. Having calculated the TPs, FPs, FNs and TNs for a threshold value, we can compute the True Positive Rate and False Negative Rate values as shown in [36].

**Fig 3:**
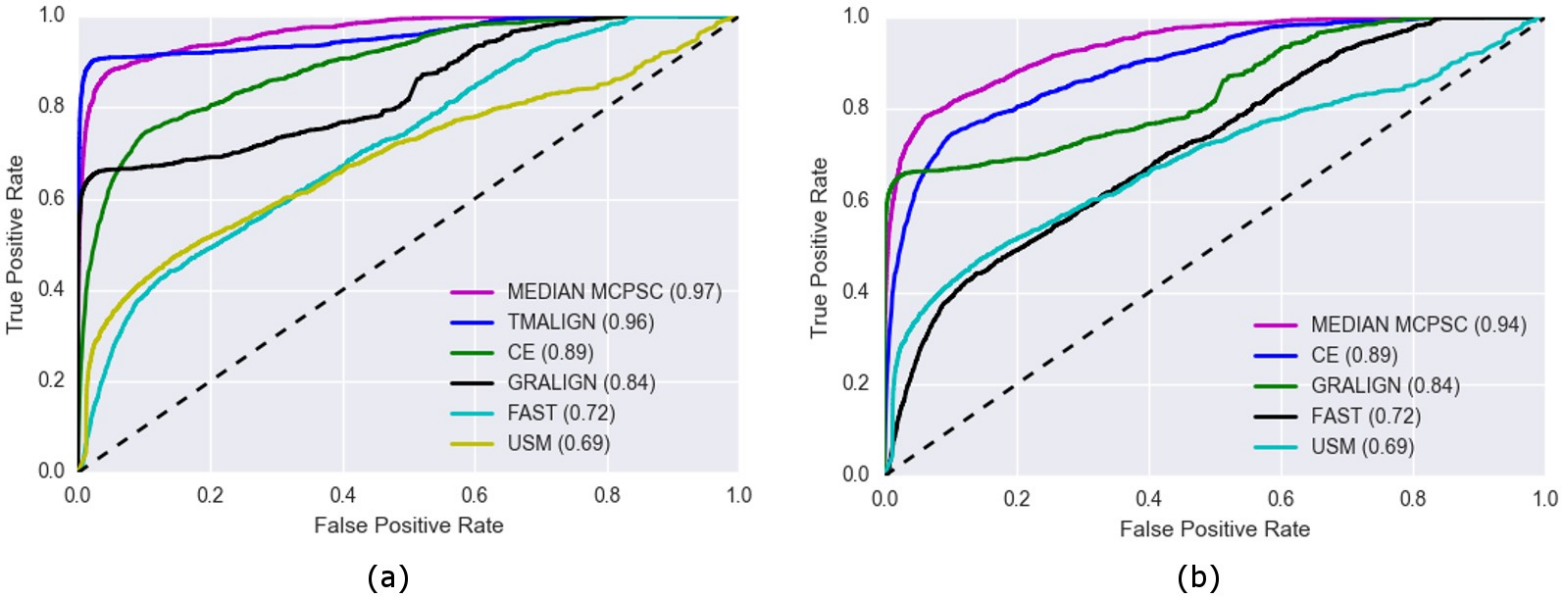
ROC curves of all PSC methods and the median MCPSC method using the Imputed dataset of pairwise similarity scores. The ROCs are generated at the SCOP Superfamily level (Level 3). Panel (a) shows the results with all five PSC methods and panel (b) with TM-align excluded.

In Fig 3(a), we see that for this dataset TM-align achieves the highest AUC among the five supported PSC methods. Moreover using the median MCPSC score matches or exceeds the AUC performance of the best component method (see also Fig C in S1 File). This actually remains the case even if we remove TM-align from the pool of the PSC methods and repeat the same analysis with the four remaining methods Fig 3(b) (see also Fig D in S1 File). In reality, we do not expect to know which PSC method will perform the best for any given dataset. So as the results suggest, combining PSC methods to obtain MCPSC scores and then using their median as the final consensus score to assess similarities is an effective strategy.

### *pyMCPSC* consensus scores can be used to accurately classify query domains

Nearest-neighbor (NN) auto-classification [37] can be used to assess how well PSC methods can classify a query domain, given pairwise PSC scores and the structural classification of other domains. When a new protein structure is determined, it is typically compared with the structures of proteins with known SCOP classifications. Therefore, the accuracy of the PSC and MCPSC based NN-classifiers effectively reflects their ability to be used for automatic protein domain classification.

Distance matrices based on the PSC and MCPSC scores are used by *pyMCPSC* to perform NN domain classification. The following process is repeated for each supported PSC and MCPSC method:

- Each domain is considered as a query and assigned the class label of its Nearest-neighbor using the pairwise scores as distances. This leave-one-out class label assignment is repeated for every domain and the predicted classes are recorded.
- The percentage of domains correctly classified is then calculated.
- A domain is correctly classified if the predicted and actual (ground truth) class labels match.

MCPSC based NN-classification matches or exceeds the performance of the best PSC method at all SCOP hierarchy levels, with and without data imputation Table 3. Moreover, whereas the classification performance of the five supported PSC component methods varies considerably for the same SCOP level, the performance of the five different MCPSC methods is consistent. This suggests that using *pyMCPSC* to implement different MCPSC methods and then using their median score in conjunction with NN classification can provide trustworthy query domain auto-classification results. These results also highlight that in the absence of ground truth information and/or lack of prior knowledge as to the best PSC method for a dataset, MCPSC can be employed to accurately auto-classify new domains.

**Table 3:** Fraction of domains correctly classified at different SCOP hierarchy levels using a Nearest-Neighbor classifier built with similarity scores produced by different PSC and MCPSC methods. In the SCOP hierarchy: Level 1 = Class, Level 2 = Fold, Level 3 = Superfamily and Level 4 = Family.

	Original dataset				Common subset				Imputed dataset			
SCOP Level	1	2	3	4	1	2	3	4	1	2	3	4
TM-align	1.00	1.00	0.99	0.99	0.74	0.57	0.57	0.57	1.00	1.00	0.99	0.99
CE	0.78	0.61	0.61	0.60	0.63	0.47	0.47	0.47	0.76	0.60	0.60	0.58
GRALIGN	1.00	1.00	1.00	1.00	0.74	0.57	0.57	0.57	0.89	0.89	0.89	0.88
FAST	0.20	0.08	0.08	0.08	0.19	0.07	0.07	0.07	0.20	0.08	0.08	0.08
USM	0.84	0.72	0.67	0.65	0.65	0.51	0.49	0.49	0.84	0.72	0.67	0.65
M1	0.99	0.98	0.98	0.98	0.73	0.57	0.56	0.56	0.99	0.99	0.98	0.98
M2	0.99	0.98	0.98	0.98	0.75	0.57	0.56	0.56	0.99	0.98	0.97	0.97
M3	1.00	1.00	1.00	1.00	0.74	0.57	0.57	0.57	1.00	1.00	1.00	1.00
M4	0.99	0.99	0.99	0.99	0.72	0.57	0.57	0.57	0.99	0.99	0.99	0.99
M5	1.00	1.00	1.00	1.00	0.74	0.57	0.57	0.57	1.00	1.00	1.00	1.00
Median MCPSC	0.99	0.99	0.99	0.99	0.74	0.57	0.57	0.57	0.99	0.99	0.99	0.99

The results show that the best MCPSC method matches the performance of the best component method and the Median MCPCS based classification is almost always optimal, which makes median MCPSC a good choice for classifying query domains when prior knowledge about the best PSC method is not available. Moreover, the performance differences of the MCPSC methods are minor, suggesting that they are all quite robust to significant variations on the performance of their component PSC methods. The lower performance observed for all methods on the Common subset is probably a result of the small percentage of domain pairs for which similarity scores are available by all methods (less than 50%).

### *pyMCPSC* reveals structural relations between domains

*pyMCPSC* uses PSC/MCPSC based distance matrices in conjunction with Multi-Dimensional Scaling (MDS) [38] to generate insightful scatterplots of protein domain organization in the structural space. An *N* × *N*, distance matrix *D* is constructed, with *N* being the number of unique domains in the dataset. Matrix element *D*_*ij*_ corresponds to 1—*S*_*ij*_, the pairwise scaled dissimilarity score of domains *d*_*i*_ and *d*_*j*_, where *i*, *j* ≤ *N*, are drawn from the imputed data set. Missing values (*N*^2^ − *P*) are set to 1. The value of 1 (max dissimilarity) is selected so that all domains appearing close in the visualization are in fact close to each other based on the selected method’s score.

**Fig 4:**
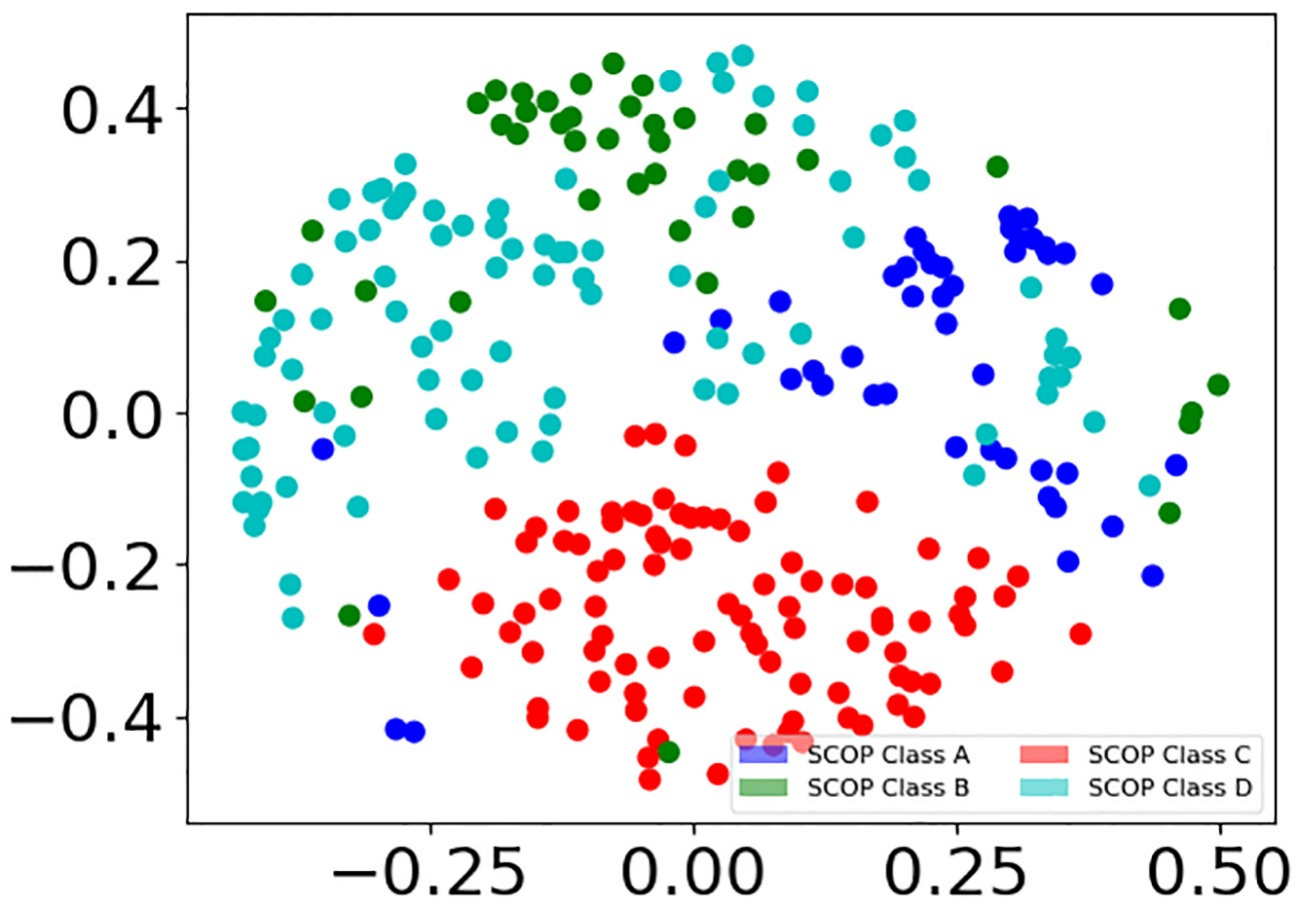
MDS scatter plot based on median MCPSC scores. Domains are colored according to their SCOP class (Level 1).

*pyMCPSC* uses matrix *D* as the basis for MDS to produce scatterplots of domains. This effectively assigns a 2-Dimensional coordinate to each protein domain constrained by the pairwise domain distances specified in matrix *D*. The resulting scatterplots can be used to visually explore a domains dataset, revealing existing correlations. Fig 4 shows the layout of the domains of the imputed dataset in 2-D space resulting from MDS using the median MCPSC scores. Such a visualization produced by *pyMCPSC* suggests that for the given dataset the SCOP Class C domains (red color) exhibit higher interdomain similarity. This is in stark contrast to the domains of SCOP Class D (cyan color) which are diffused across the scatterplot (details in Fig E in S1 File). This observation is further evidenced by the Heatmaps also generated by *pyMCPSC* (details in Figs F and G in S1 File) which in addition reveal finer level structure inside each class. The heatmaps clearly show stronger correlations between domains / folds of SCOP Class C (darker patches in the heatmaps) while no such correlations appear for other classes which justifies the observations of the scatterplots.

### *pyMCPSC* can reveal functional relations between protein domains

*pyMCPSC* uses similarity score based distance matrices (*D*) in “Phylogenetic Trees” [39] to provide functional grouping of domains. *pyMCPSC* uses a Neighbor-joining algorithm from *dendropy* [32] to create dendrograms and uses them to generate unrooted circular layout “Phylogenetic Trees”. The goal is to create trees where the domains are separated into clades based on their function [40].

**Fig 5:**
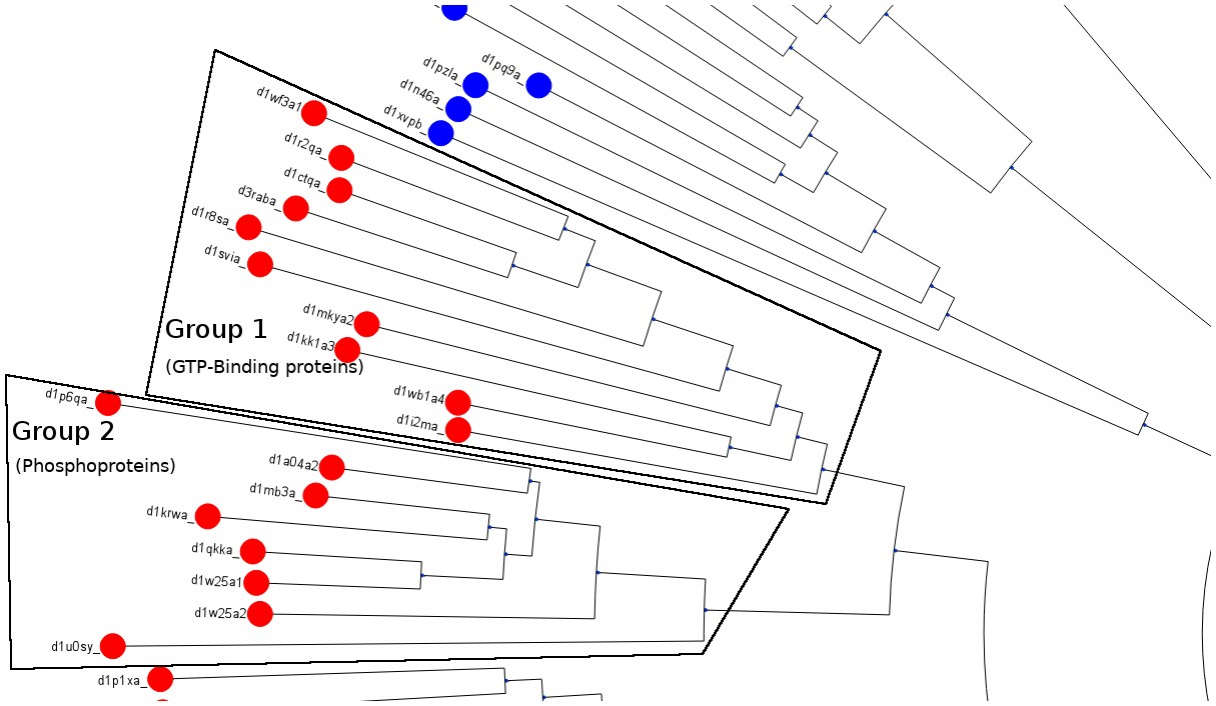
The unrooted Phylogenetic Tree based on median MCPSC consensus scores. Domains are colored according to their SCOP class (Level 1). Domains of the two clades that are marked belong to Class C but represent different functional groups (see Table C in S1 File).

In Fig 5 we have marked two groups of domains belonging to different clades in the tree. The most common keyword for Group 1 is ‘GTP-Binding’ while for Group 2 it is ‘Phosphoprotein’ (see details in Table C in S1 File). The clades of the Phylogenetic Tree generated by *pyMCPSC* could therefore be used by a researcher to identify groups of domains (within the same SCOP class as in this example) that are functionally different.

## Conclusions

We have presented a unique python based utility, called pyMCPSC that can, a) generate pairwise structure comparison and consensus scores using multiple PSC and MCPSC methods and b) use the resulting similarity scores to generate insightful visualizations to help assess structural relationships in protein datasets. Availability of utilities, such as *pyMCPSC,* will enable researchers in structural proteomics to carry out complex dataset analysis without needing to resort to distributed infrastructure scheduling. As demonstrated, *pyMCPSC* implements/supports multiple PSC and MCPSC methods, processes the pairwise PSC tasks efficiently in parallel and performs systematic structural analysis of protein domain datasets. Importantly, it is also easy to incorporate new PSC methods or implement new MCPSC in *pyMCPSC,* giving researchers a lot of flexibility with minimal effort. We intend to maintain *pyMCPSC* and extend its capabilities as needed. More flexibility will be added to the CLI providing more control over the experimental setup, such as enabling/disabling the mix of PSC methods used, configuring methods to be included in the visualizations, and improving interfaces between the modules to make *pyMCPSC* more amenable to be used as a library. We are also considering to add support in the future for returning alignments based on consensus schemes (MCPSC).

## Supporting information

S1 File Supplemental material. A document containing additional information about the software implementation (documentation, installation instructions etc,), the methods and the results presented in the manuscript.
(PDF)

S2 File Supplemental material. A document containing additional information about the software, the methods and the results generated for a very large SCOPCATH dataset. Information on the files needed and instructions on how to produce the results of the analysis are also provided.
(PDF)
