# Supplementary material for: Multi-criteria protein structure comparison and structural similarities analysis using pyMCPSC
Source: PLoS One. 2018 Oct 17;13(10):e0204587. doi: 10.1371/journal.pone.0204587 (PMC6192565; doi:10.1371/journal.pone.0204587)
Supplement: S1 File — A document containing additional information about the software implementation (documentation, installation instructions etc,), the methods and the results presented in the manuscript. (PDF) [file pone.0204587.s001.pdf]

Supplementary Material
Multi-Criteria Protein Structure Comparison and Structural Similarities Analysis using *pyMCPSC*

Anuj Sharma and Elias S. Manolakos

Contents

A Instructions for installation **2**

A.1 Supported PSC methods . . . 2

A.2 Installation . . . 3

A.3 Extending *pyMCPSC* . . . 3

A.3.1 Template for adding new PSC method wrappers . . . 4

B Supplementary Methods **6**

B.1 Protein holdings in Protein Data Bank . . . 6

B.2 Supervised learning of weights . . . 7

C Supplementary Results **8**

C.1 ROC Analysis . . . 8

C.2 Extended Nearest-Neighbor classification analysis . . . 11

C.3 Multidimensional Scaling Scatterplots of protein domains . . . 11

C.4 Similarity Based Heatmaps at Domain and Fold Levels . . . 11

C.5 Similarity Based Unrooted Phylogenetic Trees . . . 16

A Instructions for installation

The choice of the Python programming language to implement *pyMCPSC* was inspired by its: a) popularity, b) ease of extensibility and c) good support for text processing. *pyMCPSC* uses the Python *multiprocessing* module to spawn threads, each one executing an external binary using the *subprocess* module [1]. Python and its extensive libraries are almost ubiquitously available making *pyMCPSC* suitable for a diverse number of platforms. The limiting factor is the availability of the component PSC method binaries, or the availability of the source code to compile them for different platforms (currently *pyMCPSC* has been tested only on machines running 64-Bit Linux O/Ss). *pyMCPSC* has been tested on Python 2 (version 2.7) and Python 3 (version 3.5).

A.1 Supported PSC methods

The current version of *pyMCPSC* contains wrappers for five well known pairwise Protein Structure Comparison (PSC) methods: a) *CE* [2], b) *TM-align* [3], c) *FAST* [4], d) *GRALIGN* [5] and e) *USM* [6]. These implementations can serve as examples of how to quickly extend the utility with more PSC methods, as their binaries become available in the future. Download links for software corresponding to these methods are listed in Table A. USM is not included in the Table because, the program is not available for download, the contact map generation binary of GRALIGN is reused and the standard Python compression libraries are used for generating the similarity scores.

**PSC Method**	**Download URL**
CE	http://source.rcsb.org/jfatcatserver/ceHome.jsp
TM-align	http://zhanglab.ccmb.med.umich.edu/TM-align/
GRALIGN	http://www0.cs.ucl.ac.uk/staff/natasa/GR-Align/index.html
FAST	https://biowulf.bu.edu/FAST/download.htm

Table A: Download links for PSC methods used in *pyMCPSC*.

The user may use the *scripts/psc_get.sh* script packaged with *pyMCPSC* to download the source and binaries of the default PSC methods. Where sources are available, the script downloads and builds the sources on the user's machine. We note that sources for all the programs are not available - FAST and GRALIGN are available only in Binary formats. The script uses several standard Linux tools and requires various compilers during the process. As as a first step the script checks for availability of the dependencies and exits if a dependency is missing. It is beyond the scope of these instructions to direct the user on how the missing dependency may be resolved. The final binaries of the PSC methods are placed in the *programs* folder created where the script is executed.

A.2 Installation

The current implementation of *pyMCPSC* has been built and tested on a machine running 64-bit Linux. We also provide a pre-built docker image (available for download from http://bit.ly/2lRj7xD) which has been tested on multiple operating systems (including Mac OS and Windows). Detailed build and usage instructions can be found in the documentation of *pyMCPSC* (available for download from http://bit.ly/2xdP21j). The documentation can be generated from source on any system with the Make toolchain and Sphinx setup. The program parameters can be specified on the CLI (Figure A). *pyMCPSC* provides a set of sensible default fallback values for the optional arguments. Descriptions and default values for all CLI arguments are provided in *pyMCPSC* documentation. If no values are specified by the user *pyMCPSC* runs the experiment described in this paper. Results (including figures) generated by *pyMCPSC* are placed in directories located in the current working directory (CWD), i.e. the one from where the program is launched.

A.3 Extending *pyMCPSC*

The functionality of *pyMCPSC* can be extended by including more PSC methods in the analysis as described below. In order to introduce a new PSC method into the processing pipeline, a new *Class* must be added to the utility. The *Classes* provide functionality to run

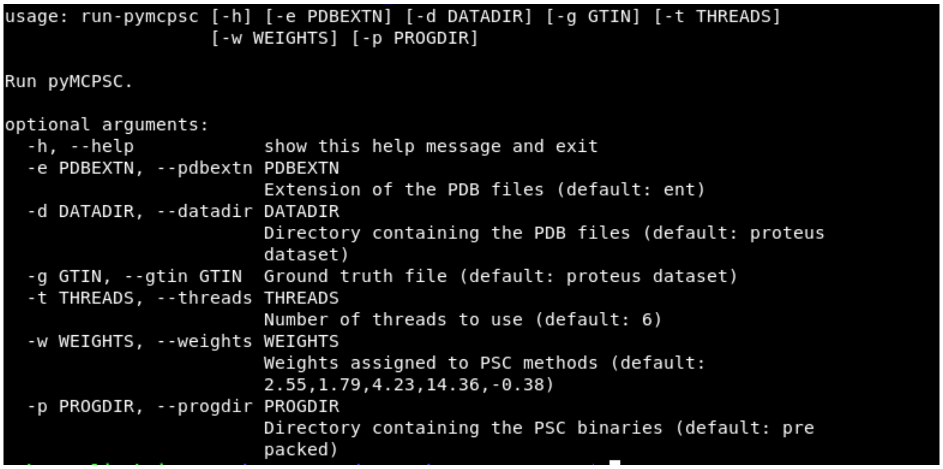

Figure A: Usage help message print out by *pyMCPSC* explaining the parameters accepted by the program.

the external PSC binary and provide a text level interface, between the utility and the PSC binary, to read the output generated by the PSC method. Once this key implementation has been written, minor edits to other sections of the code are sufficient to include the method into the processing. The implementations of the 5 PSC wrapper classes included in *pyMCPSC* by default provide examples of how a new wrapper class may be written. It must be noted that the *pyMCPSC* does not implement PSC methods itself, but rather expects to be provided with executable binaries (one per component PSC method participating in the MCPSC scores calculation). A wrapper class must be written to allow the external binary to be usable in *pyMCPSC*.

A.3.1 Template for adding new PSC method wrappers

Extending *pyMCPSC* to incorporate a new PSC method requires implementing a class that follows the template shown in Listing 1 below. The key aspects of the template are: a) the path to the binary must be passed at instantiation time, and b) the results of pairwise comparison must be returned for each pair so that they can be used in *pyMCPSC*.Examples of implementing this template for a PSC method can be found in the file *run.py* in *pyMCPSC* sources.

The following steps must be followed in order to introduce a new PSC method to *pyMCPSC* and make its processing results for available for the consensus scores calculation:

- Implement a class based on the template to handle the input and output of the PSC method
- Instantiate the class and pass it to the doMulti method defined in *run.py* (see line 476 for an example)
- Add the method entry to the name lists *psc_methods, psc_method_names* defined in *run_mcpsc.py*
- Add the output file generated by the execution of the PSC method to the *infiles* list defined in the file *postprocessing.py* and update the column names for the output file generated by the module
- Add the method weight for consensus scores calculation by updating the value of *__def_WEIGHTS__* in *run_pymcpsc.py*

```python
class PSC_HANDLER:
  def __init__(self, path_to_binary):
    # store path in class instance and
    # perform any additional house keeping

  def process_pair(self, domain1, domain2):
    # execute external binary with domains
    ...
    # read execution output
    ...
    # collect results of pairwise processing
    ...
    return results
```

Listing 1: Template of *Class* that needs to be added to our command-line utility to introduce processing for a new PSC method. An instance of this class can then be passed to a thread pool for distributed processing of a list of pairwise PSC jobs.

B Supplementary Methods

B.1 Protein holdings in Protein Data Bank

The number of protein structures catalogued in the Protein Data Bank (PDB) has grown exponentially over the last few years as noted in Figure B.

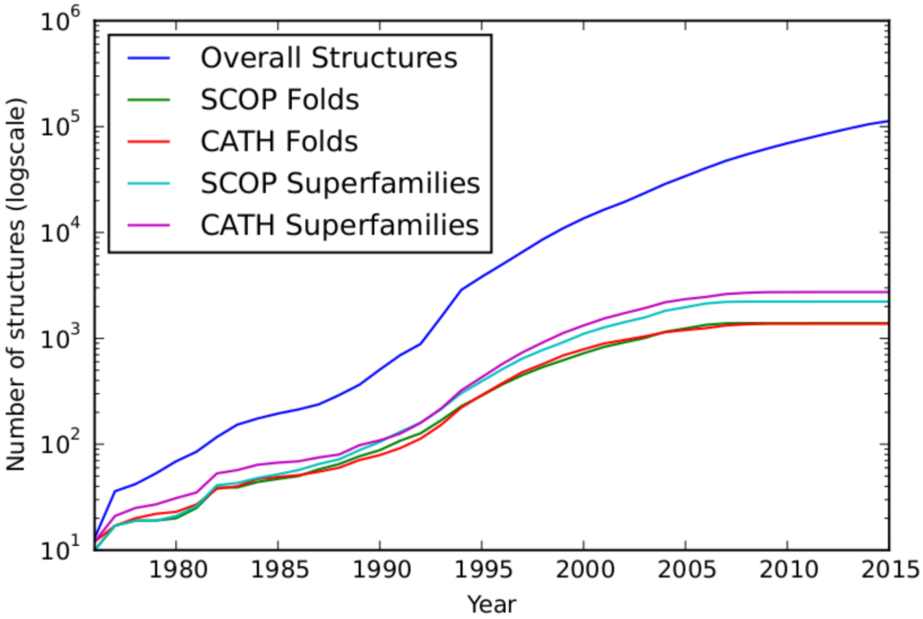

Figure B: Statistics of protein holdings in Protein Data Bank (PDB), data taken from RCSB PDB. The data for Year 2015 is incomplete.

B.2 Supervised learning of weights

In order to learn the weights for the supported PSC methods using supervised learning the following steps were undertaken:

- Read pairwise PSC scores generated by $M = 5$ PSC methods for all domain pairs. Each pair is represented by a feature vector containing all PSC method scores.
- Each pair is categorized as belonging to Class 1 - meaning pairs from the same SCOP classification - or Class 0 - meaning pairs belonging to different SCOP classification.
- Perform 10-fold cross-validation with Unimputed (intersection) and Imputed datasets evaluating the performance of a binary (0/1) Logistic Regression (LR) model.
- 3 metrics - Sensitivity (Recall), Specificity and Precision are recorded at each iteration (10 in all).

This cross validation procedure was repeated several times, each time using a different percentage (varied from 1% to 100%) of the full data used to learn the coefficients. The

lowest percentage for which the learnt model performed well on the 3 metrics for the Proteus domains dataset was 10%.

C Supplementary Results

We present here results that pertain to the experimental setup with the Proteus_300 dataset [7]. As expected, not all domain pairs were successfully processed by every PSC method. Missing PSC scores for pairs of domains is an inevitable problem when processing sizeable datasets, due to the third party binaries incorporated in the utility for PSC methods or PDB file errors. This is especially evident in large scale data analysis where it may not be possible to determine and fix completely the exact causes of the problem.

C.1 ROC Analysis

In Figure C we provide the ROC curves and corresponding AUC values for all PSC and the Median MCPSC method for all three datasets of domain pairs as defined in the manuscript. In general, the closer a ROC curve follows the left-side border and then the top border of the ROC space, the better the classification performance of the corresponding PSC method, because it indicates that high PSC scores (i.e. high similarity) are assigned to domain pairs where both domains belong to the same SCOP Superfamily (Scop Level 3). The results show that for this dataset TM-align happens to be the best performing PSC method. However, the median MCPSC matches or exceeds the AUC of the best component PSC method in all cases. Figure D shows the ROC curves comparing the Median MCPSC and the PSC methods after excluding TM-align. The consensus scoring scheme outperforms against its component methods.

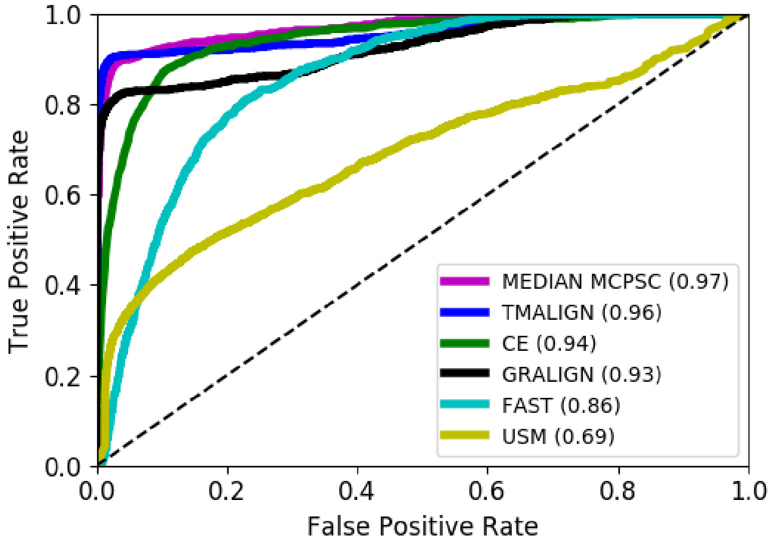

(a) Original Dataset

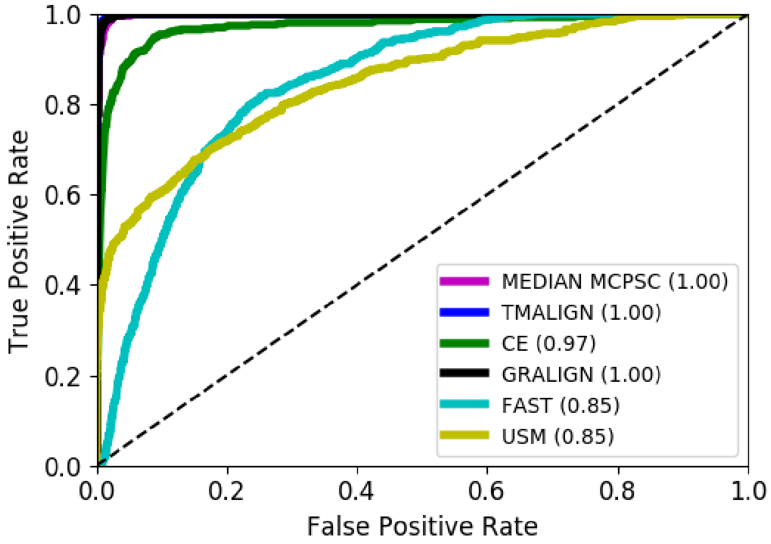

(b) Common Dataset

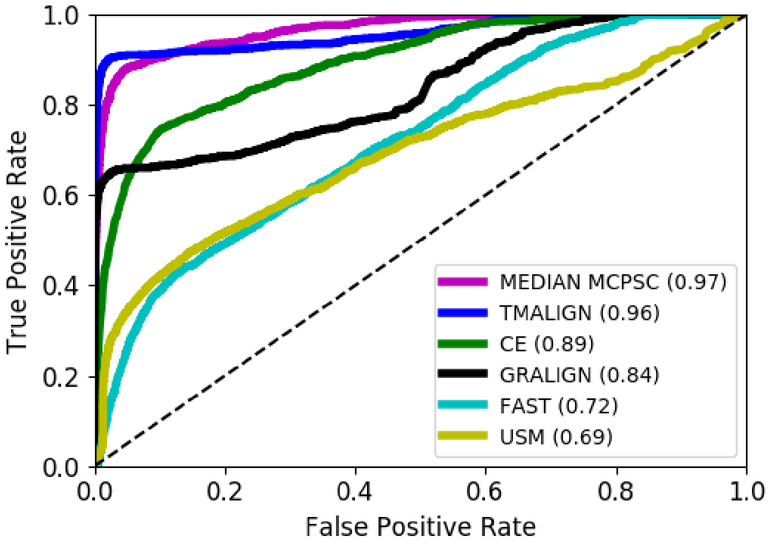

(c) Imputed Dataset

Figure C: ROC plots generated by *pyMCPSC*. The panels are plots for the PSC methods and median MCPSC method over the three variations of the domain pairs Proteus 300 dataset. The Area Under the Curve (AUC) is provided in parentheses. The ROCs are generated at the SCOP Superfamily level (Level 3).

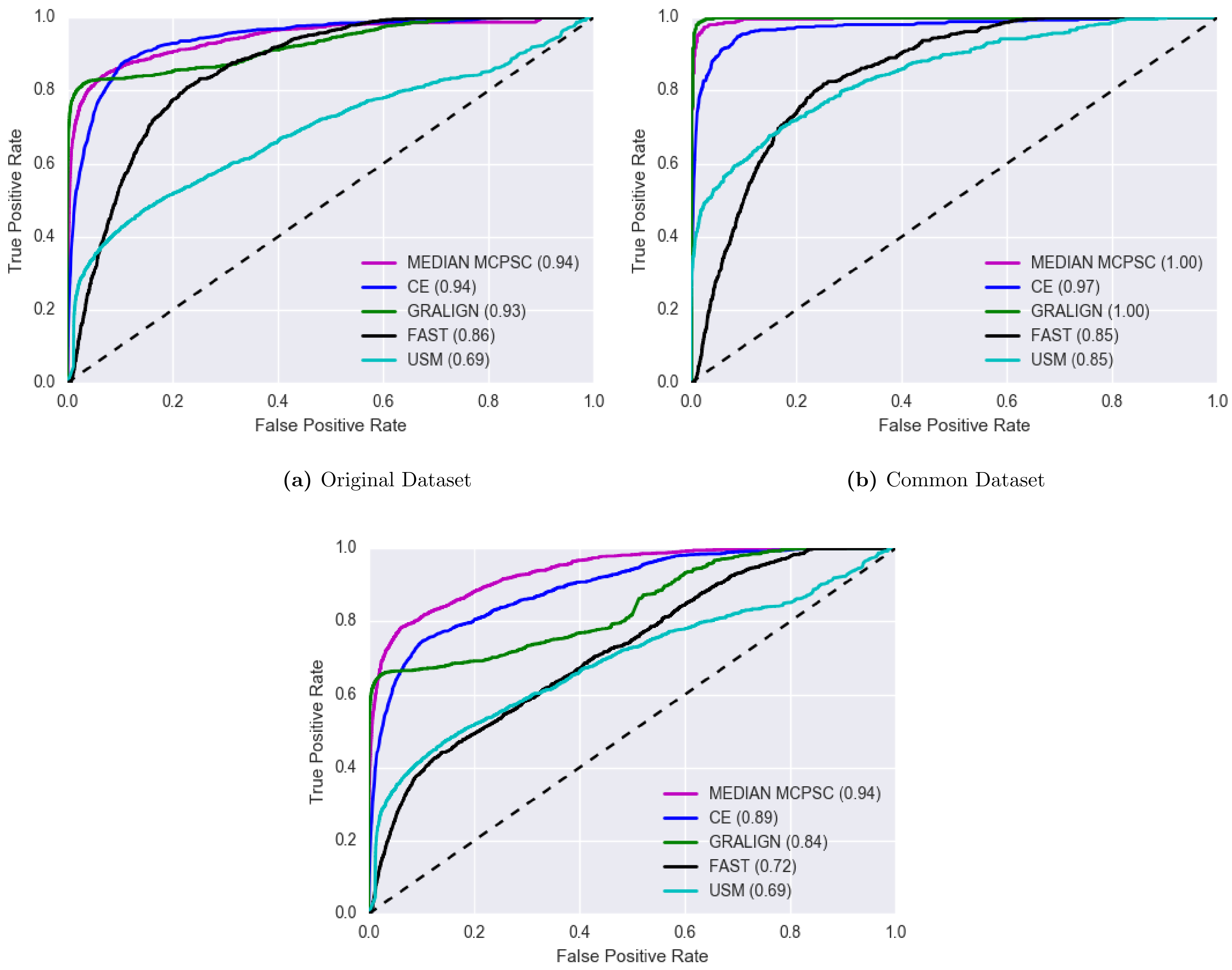

(a) Original Dataset

(b) Common Dataset

(c) Imputed Dataset

Figure D: Median MCPSC matches or exceeds the best performing method (CE) among the remaining four component PSC methods after removing TM-align from the pool used to derive the MCPSC consensus scores. The ROCs are generated at the SCOP Superfamily level (Level 3).

C.2 Extended Nearest-Neighbor classification analysis

Using the data generated by *pyMCPSC* (stored in file processed.imputed.mcpsc.csv) a break down of the performance of the different PSC and Median MCPSC method was generated, as shown in Table B below. In addition to the data generated by *pyMCPSC* functional annotation of the SCOP families were obtained from (http://scop.berkeley.edu) in order to assess if there is a correlation between the classification performance and the domain SCOP families. It is interesting to observe that there is no component method that is uniformly best on all families. Moreover, the consensus Median MCPSC classifier performs best for most families, with TM-align exceeding its performance on very few cases, however there is no pattern to these families. Interestingly, purely RMSD based methods (CE and Fast) are the least effective for most Folds. Further, we compared the domains misclassified by the different PCS/MCPSC method however there appeared to be no correlation between the misclassified domains.

C.3 Multidimensional Scaling Scatterplots of protein domains

In Figure E we provide the Multi-dimensional Scaling based scatterplot visualizations generated using distance matrices based on the scaled dissimilarity scores of the different PSC methods. The scatter plots of the PSC methods differ significantly.

C.4 Similarity Based Heatmaps at Domain and Fold Levels

Heatmaps provide further evidence for the grouping of Class domains observed in the MDS based scatter plots. *pyMCPSC* uses similarity score based distance matrices in conjunction with Heatmaps. An $N \times N$ matrix, $\mathbf{S}$, is generated where $\mathbf{S}_{ij}$ is the pairwise similarity score of domains d_i and d_j, $i, j \leq N$, using the imputed data set. This similarity matrix is used to generate the Domain level heatmaps as shown in Figure E for median MCPSC scores. Similarly, a $T \times T$ matrix, $\mathbf{F}$ can be constructed for a selected method, with T being the number of unique SCOP Folds in the dataset. Element $\mathbf{F}_{ij}$ of this matrix corresponds to the mean pairwise scaled similarity score of domains belonging to folds t_i and t_j, where $i, j \leq T$.

Each element of this matrix therefore effectively represents the similarity between folds as an average of the similarity between their domains. This similarity matrix is used to generate the Fold level heatmaps as shown in Figure F for median MCPSC scores. *pyMCPSC* saves the heatmap matrices to the *outdir* as CSV files allowing the visualization to be generated using other third-party tools if needed. When the size of the matrices **S** or **F** is larger than 300×300, *pyMCPSC* disables image generation to avoid potentially creating huge files.

Median MCPSC	TM-align	CE	FAST	GRALIGN	USM	# Domains	SCOP Family	Functional Annotation
1.00	1.00	0.13	0.00	1.00	0.63	8	c.67.1.1	AAT-like
1.00	0.89	0.22	0.00	1.00	0.11	9	c.1.10.1	Class I aldolase
1.00	1.00	0.50	0.10	0.90	0.80	10	d.131.1.2	DNA polymerase processivity factor
1.00	1.00	0.40	0.00	1.00	1.00	10	a.45.1.1	Glutathione S-transferase (GST), C-terminal domain
1.00	1.00	0.50	0.00	0.90	0.50	10	c.94.1.1	Phosphate binding protein-like
1.00	1.00	1.00	0.10	0.90	0.90	10	a.25.1.1	Ferritin
1.00	1.00	1.00	0.00	1.00	0.86	7	b.36.1.1	PDZ domain
0.89	1.00	0.33	0.00	0.89	0.78	9	d.54.1.1	Enolase N-terminal domain-like
1.00	1.00	0.56	0.00	0.89	0.78	9	d.108.1.1	N-acetyl transferase, NAT
1.00	1.00	0.88	0.00	1.00	0.88	8	d.15.1.1	Ubiquitin-related
0.90	1.00	0.30	0.00	0.90	0.80	10	d.144.1.7	Protein kinases, catalytic subunit
1.00	1.00	0.14	0.00	1.00	0.71	7	a.104.1.1	Cytochrome P450
1.00	1.00	0.00	0.00	1.00	0.30	10	c.1.8.3	beta-glycanases
1.00	1.00	0.11	0.00	1.00	0.78	9	c.93.1.1	L-arabinose binding protein-like
1.00	1.00	1.00	0.11	0.89	1.00	9	d.162.1.1	Lactate and malate dehydrogenases, C-terminal domain
1.00	1.00	0.60	0.00	0.90	0.60	10	c.37.1.20	Extended AAA-ATPase
1.00	1.00	0.60	0.80	1.00	1.00	10	c.2.1.5	LDH N-terminal domain-like
1.00	1.00	1.00	0.13	0.88	0.75	8	c.23.1.1	CheY-related
1.00	1.00	0.80	0.00	0.20	0.00	10	d.153.1.4	Proteasome subunits
1.00	1.00	0.56	0.00	1.00	0.44	9	c.2.1.2	Tyrosine-dependent oxidoreductases
0.89	1.00	0.89	0.22	0.89	0.78	9	d.58.7.1	Canonical RBD
1.00	1.00	0.80	0.30	0.70	0.70	10	d.169.1.1	C-type lectin domain
1.00	1.00	0.78	0.00	0.78	0.78	9	a.1.1.2	Globins
1.00	1.00	0.83	0.50	1.00	1.00	6	d.58.17.1	HMA, heavy metal-associated
1.00	1.00	0.63	0.00	0.50	0.50	8	b.1.2.1	Fibronectin type III
1.00	1.00	0.90	0.00	0.70	0.40	10	b.69.4.1	WD40-repeat
1.00	1.00	0.50	0.40	1.00	0.60	10	c.37.1.8	G proteins
1.00	1.00	0.90	0.00	0.40	0.50	10	b.1.1.2	C1 set domains (antibody constant domain-like)
0.89	1.00	0.00	0.00	0.89	0.44	9	a.123.1.1	Nuclear receptor ligand-binding domain
0.86	0.86	0.71	0.00	0.71	0.29	7	b.1.1.4	I set domains

Table B: Fraction of domains correctly classified at SCOP family level using a Nearest-Neighbor classifier built with similarity scores produced by different PSC and MCPSC methods.

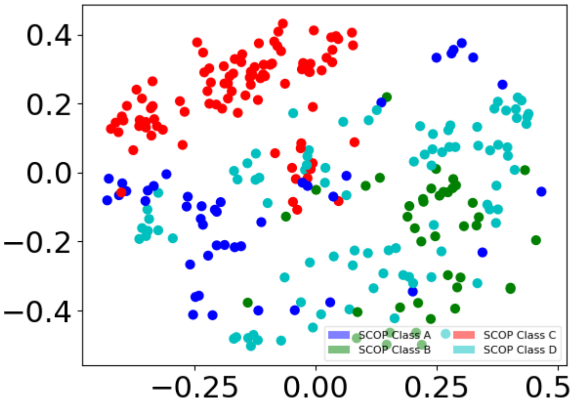

(a) CE

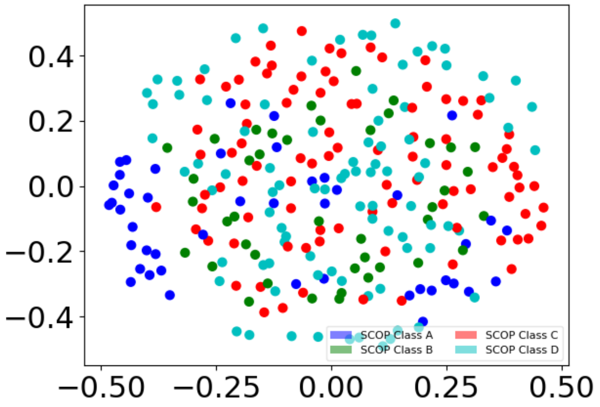

(b) FAST

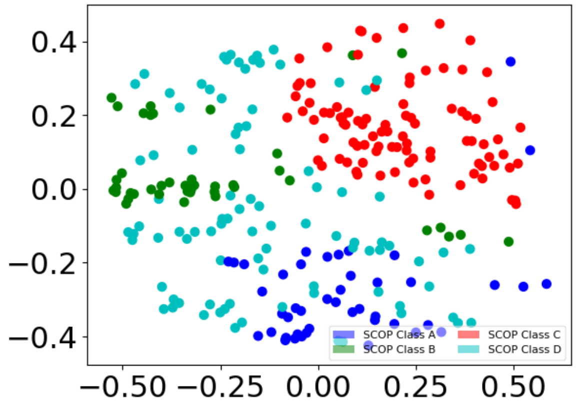

(c) TM-align

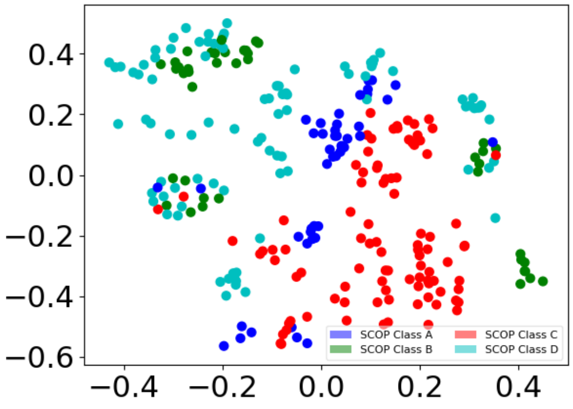

(d) GRALIGN

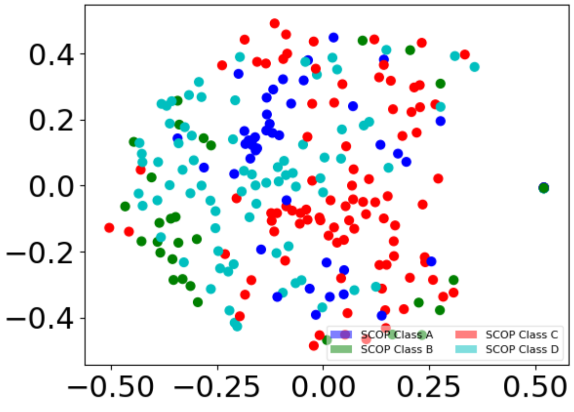

(e) USM

Figure E: MDS scatterplots for the five PSC methods generated using distance matrices and the imputed dataset. The points are colored by the ground-truth SCOP Level 1 classification of each domain. Blue = SCOP Class A, Green = SCOP Class B, Red = SCOP Class C and Cyan = SCOP Class D.

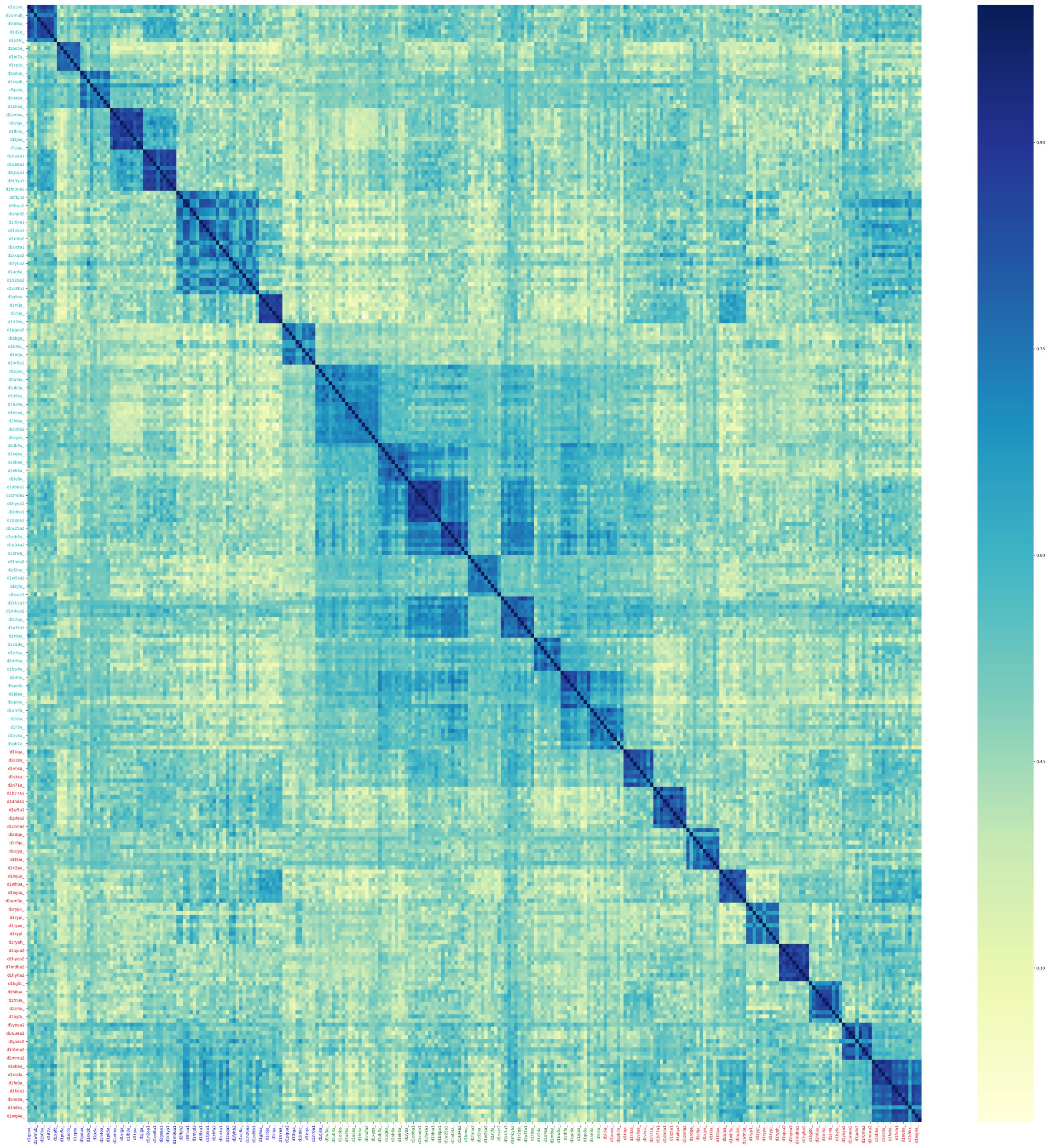

Figure F: Heatmaps generated for the median MCPSC method using similarity matrices at the Domain level for the imputed dataset. The domains are colored according to their ground-truth SCOP classification. Blue = SCOP Class A, Green = SCOP Class B, Red = SCOP Class C and Cyan = SCOP Class D. Heatmaps reveal presence of sub-clusters of domains within each SCOP class evidenced by darker regions of varying sizes along the main diagonal.

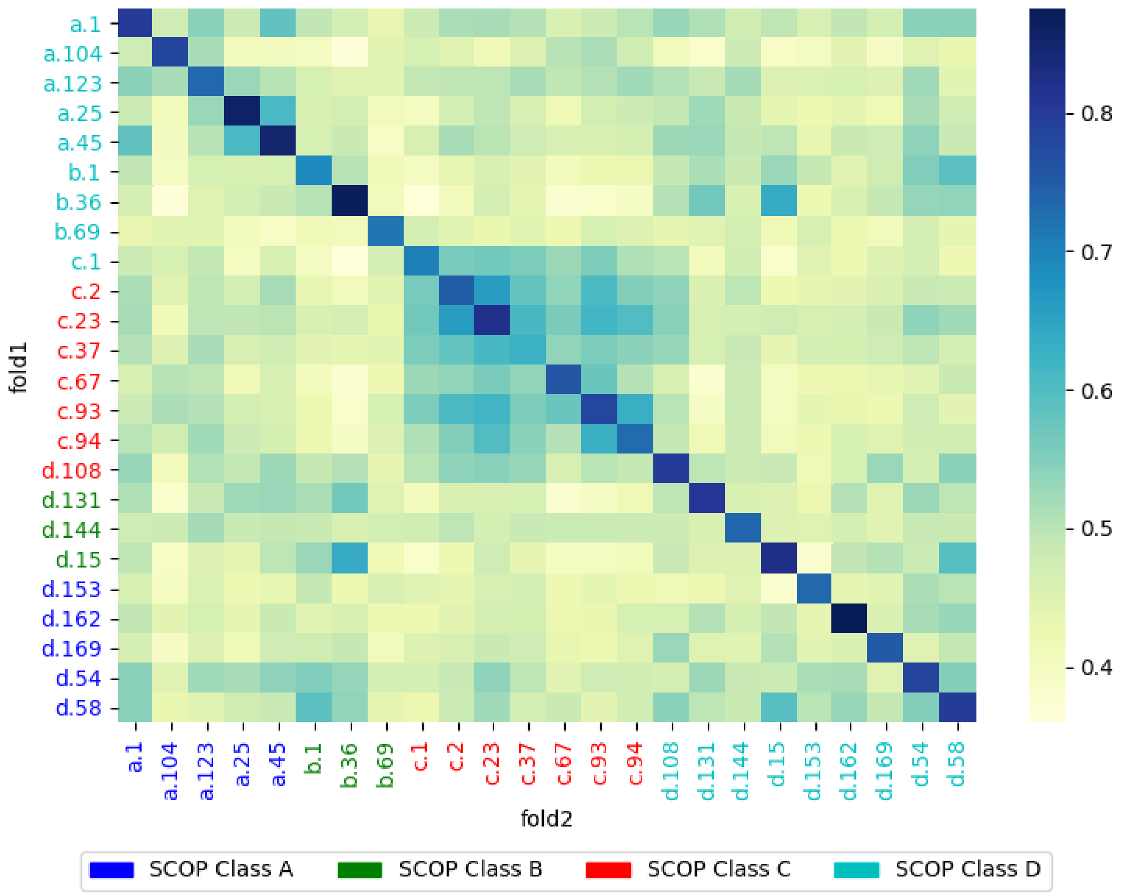

Figure G: Heatmaps generated for the median MCPSC method using similarity matrices at the Fold level for the imputed dataset. The folds are colored according to the ground-truth SCOP classification. Blue = SCOP Class 'a', Green = SCOP Class 'b', Red = SCOP Class 'c' and Cyan = SCOP Class 'd'. Heatmaps reveal presence of sub-clusters of folds within each SCOP class especially for SCOP Class 'c'.

C.5 Similarity Based Unrooted Phylogenetic Trees

We can use *pyMCPSC* to generate an unrooted 'Phylogenetic Tree' visualization of the dataset domains. A small part of an unrooted Phylogenetic tree constructed is shown in Fig 5 of the manuscript (the complete Tree shown in Fig H below). Two Clades were selected for comparison that are at the same level in the tree and contain domains from the same SCOP Class. The goal was to assess the potential difference between the domains in the

two clades in terms of their biological function. In order to obtain information about the function of the domains the following process was repeated:

- Download pdb-to-swissprot data from: http://www.uniprot.org/docs/pdbtosp
- Download uniprot descriptions from: http://uniprot.org
- Find keywords applicable to each domain (pdb - swissprot number - keyword)

At the end of this process we have obtained all keywords associated with each domain of the dataset. The most frequent keyword occurring in the domains of each clade was then determined. Clade 1 consists of 9 domains: d1wf3a1, d1r2qa_, d3raba_, d1ctqa_, d1r8sa_, d1svia_, d1mkya2, d1kk1a3, d1i2ma_ and Clade 2 consists of 4 domains: d1a04a2, d1w25a1, d1qkka_, d1w25a2 (domains for which no keywords were found were excluded). Table C lists the frequencies of the keywords for both clades. As can be seen domains of Clade 1 are G-protein regulators while Clade 2 domains are Phosphoproteins.

**Clade 1**		**Clade 2**	
**Function**	**Count**	**Function**	**Count**
GTP-binding	9	Phosphoprotein	4
Nucleotide-binding	9	Nucleotide-binding	4
Complete proteome	9	Complete proteome	4
3D-structure	9	Two-component regulatory system.	4
Reference proteome	8	3D-structure	4
Membrane	5	Cytoplasm	3
Cytoplasm	5	Transcription	2
Cell membrane	5	Transferase	2
Lipoprotein	4	Reference proteome	2
Protein transport	4	Activator	2
Transport.	3	GTP-binding	2
Direct protein sequencing	3	Repeat	2
Prenylation	3	Transcription regulation	2
Acetylation	3	DNA-binding	2
Golgi apparatus	2	Cell cycle	2
Cell division	2	Metal-binding	2
Alternative splicing	2	ATP-binding	2
Phosphoprotein	2	Magnesium	2
Nucleus	2	Differentiation	2
Cell cycle	2	Transducer	2

Methylation	2	Nitrate assimilation	1
Polymorphism	1	Plasmid	1
Cytoplasmic vesicle	1	Repressor	1
ER-Golgi transport	1		
Disease mutation	1		
Septation.	1		
Cell inner membrane	1		
Ribosome biogenesis	2		
Metal-binding	1		
rRNA-binding.	1		
Proto-oncogene	1		
Ubl conjugation.	1		
Exocytosis	1		
Host-virus interaction	1		
Synaptosome	1		
Synapse	1		
Initiation factor	1		
Endocytosis	1		
S-nitrosylation.	1		
Magnesium	1		
Protein biosynthesis.	1		
Postsynaptic cell membrane	1		
RNA-binding	1		
Endosome	1		
Phagocytosis	1		
Myristate	1		
Palmitate	1		
Isopeptide bond	1		
Mitosis	1		
Repeat	1		
Cell projection	1		
Cell junction	1		
Transport	1		

Table C: Frequency of functional keywords obtained for the domains of the two Clades identified on the Phylogenetic Tree. Keywords are obtained from Uniprot by matching the domain names with corresponding Uniprot Ids.

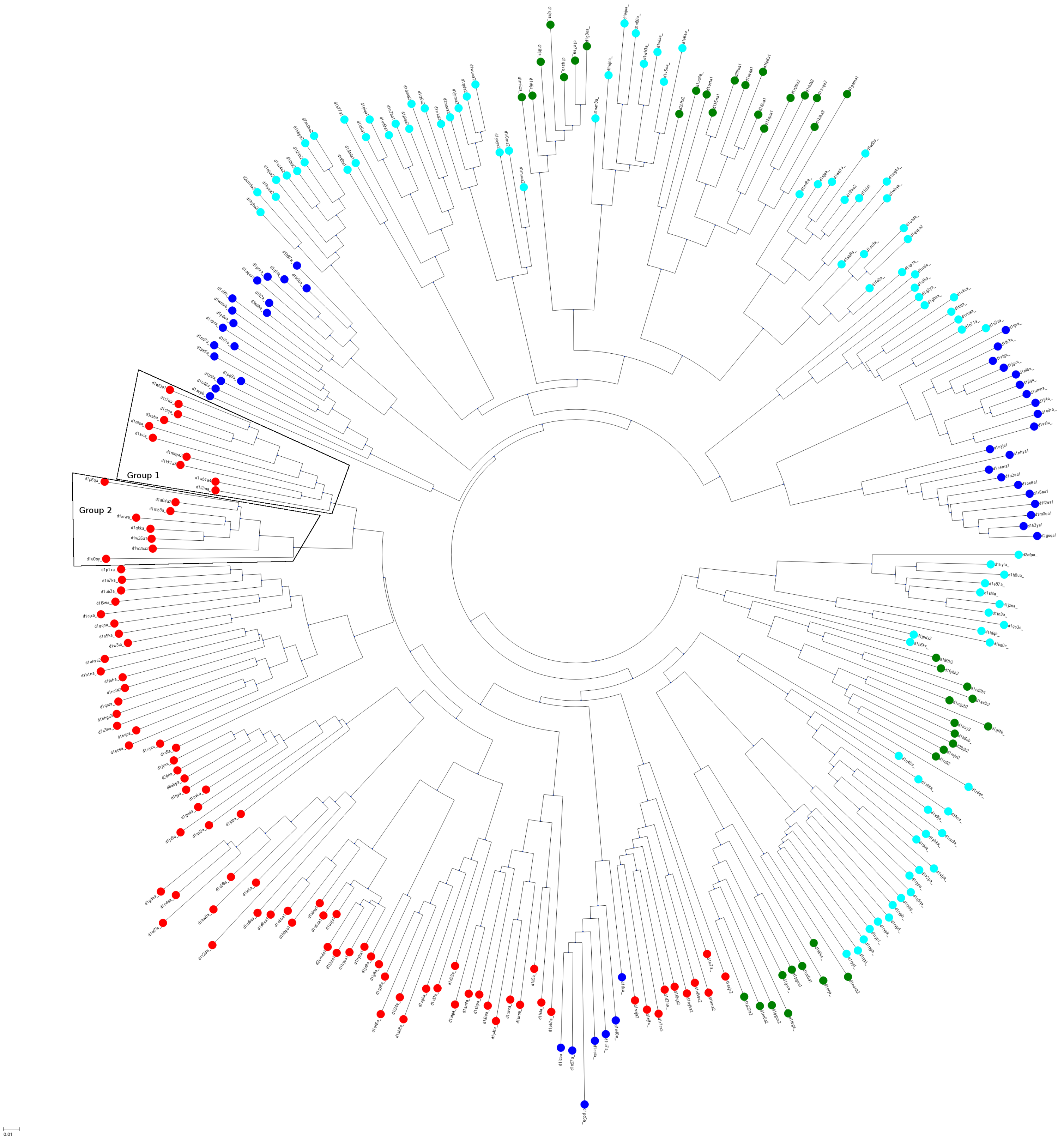

Figure H: The unrooted 'Phylogenetic Tree' based on median MCPSC consensus scores. Domains are colored according to their SCOP class (Level 1). Domains of both clades circled belong to Class C but represent different functional groups.

References

[1] Guido Van Rossum. Python tutorial, Technical Report CS-R9526. Technical report, Centrum voor Wiskunde en Informatica (CWI), Amsterdam, May 1995.

[2] I N Shindyalov and P E Bourne. Protein structure alignment by incremental combinatorial extension (CE) of the optimal path. *Protein Engineering*, 11(9):739–747, 1998.

[3] Yang Zhang and Jeffrey Skolnick. TM-align: a protein structure alignment algorithm based on the TM-score. *Nucleic Acids Research*, 33(7):2302–2309, 2005.

[4] Jianhua Zhu and Zhiping Weng. FAST: a novel protein structure alignment algorithm. *Proteins*, 58(3):618–627, 2005.

[5] Noel Malod-Dognin and Natasa Przulj. Gr-align: fast and flexible alignment of protein 3d structures using graphlet degree similarity. *Bioinformatics*, 30(9):1259–1265, 2014.

[6] N. Krasnogor and D. A. Pelta. Measuring the similarity of protein structures by means of the universal similarity metric. *Bioinformatics*, 20(7):1015–1021, May 2004.

[7] Rumen Andonov, Nicola Yanev, and Noël Malod-Dognin. *An Efficient Lagrangian Relaxation for the Contact Map Overlap Problem*, pages 162–173. Springer, 2008.
