# Supplementary material for: Multi-criteria protein structure comparison and structural similarities analysis using pyMCPSC
Source: PLoS One. 2018 Oct 17;13(10):e0204587. doi: 10.1371/journal.pone.0204587 (PMC6192565; doi:10.1371/journal.pone.0204587)
Supplement: S2 File — A document containing additional information about the software, the methods and the results generated for a very large SCOPCATH dataset. Information on the files needed and instructions on how to produce the results of the analysis are also provided. (PDF) [file pone.0204587.s002.pdf]

Supplementary Material
Multi-Criteria Protein Structure Comparison and Structural Similarities Analysis using *pyMCPSC* - Analysis using a large benchmark dataset

Anuj Sharma and Elias S. Manolakos

Contents

A Dataset **2**

A.1 Acquiring the dataset 2

B Results **2**

B.1 ROC Analysis 3

B.2 Nearest-Neighbor classification 3

B.3 Multidimensional Scaling Scatterplots of protein domains 6

A Dataset

The Gold-standard benchmark dataset introduced in [1] was used in this work. The dataset contains protein domains that are consistently defined in both SCOP v1.75 and CATH v3.2.0 (i.e. with domain overlap greater than 80%) and that share less than 50% of sequence identity. Further, the benchmark only considers domain pairs that are consistently classified across the SCOP fold classification and the CATH topology classification. The dataset consists of $N = 6759$ unique domains and defines $P =$ 3,213,631 domain pairs (similar and non-similar sets combined) [1]. Further, the 6759 domains are classified into 11 (4) Classes, 792 (780) Folds and 1348 (1550) Superfamilies according to the SCOP (CATH) classification databases respectively.

A.1 Acquiring the dataset

- Download and unpack the similar and non-similar pairs files from the SCOP-CATH dataset downloads page `https://drupal.bio.ifi.lmu.de/SCOPCath/`
- Create a unique list of domains in the files and use the *download.sh* file (in *pyMCPSC* sources) to download the PDB structures (details in *pyMCPSC* documentation).
- Rename the combined file (with the domain pairs) as the ground-truth when running *pyMCPSC* (the file is already in the correct format to be used as ground truth).

A zip file containing the ground truth, domains list and instructions for acquiring all the data required to run pyMCPSC on the SCOPCATH dataset can be downloaded from http://bit.ly/2tXYhQP.

B Results

As expected, not all domain pairs were successfully processed by every PSC method. Missing PSC scores for pairs of domains is an inevitable problem when processing sizeable datasets, due to the third party binaries incorporated in the utility for PSC methods, or PDB file

errors. It must be noted that due to the very large size of the dataset ($>$ 300 domains) heatmaps and phylogenetic tree image generation is disabled by default in *pyMCPSC*.

B.1 ROC Analysis

Figure A shows the Receiver Operating Characteristic (ROC) curves [2] for the PSC methods and the median MCPSC taken over the entire dataset of domain pairs (3,213,631). It can be seen that the Median MCPSC performs as well its component PSC methods in the scenario where PSC scores from all methods are available for all domain pairs. In general, the closer the curve follows the left-hand border and then the top border of the ROC space, the better the performance of the PSC method because it indicates that low PSC scores (i.e. low dissimilarity) are assigned to domain pairs where both domains belong to the same CATH class.

B.2 Nearest-Neighbor classification

The performance of Nearest-Neighbor classifiers built using the pairwise similarity scores are summarized in Table A. The table shows the performance of the Nearest-Neighbor (NN) classification with the three datasets (details on page 7 of the manuscript). The results show that the best MCPSC method (M5) matches the performance of the best component method. This is likely because in M5 (user defined weights) the individual PSC method weights were assigned by supervised training (page 7, Supplementary File 1). As suggested also by the ROC curves, Median MCPSC based classification is performing consistently very well, which makes median MCPSC a good choice for classifying query domains when prior knowledge about the best performing PSC method is not available, as usually the case. It must be noted that the Median MCPSC classification performance is not the median of the five MCPSC methods, but rather the performance of the NN-classifier built using the median of the MCPSC pairwise similarity scores.

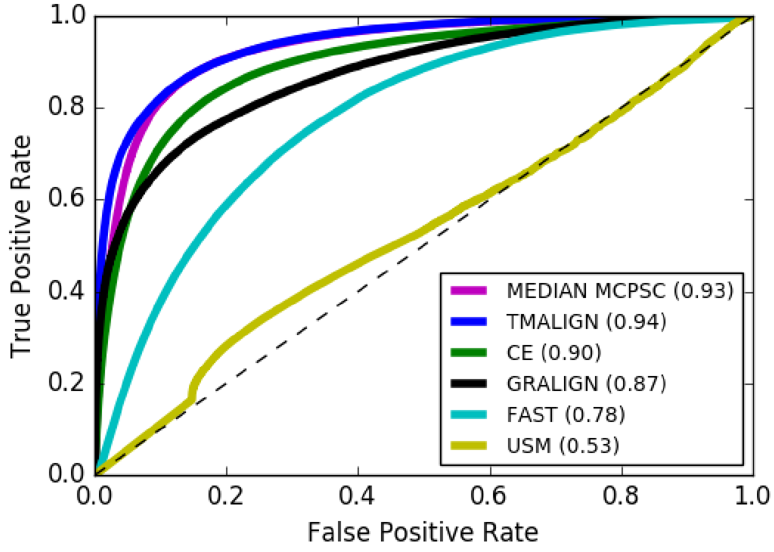

(a) Original Dataset

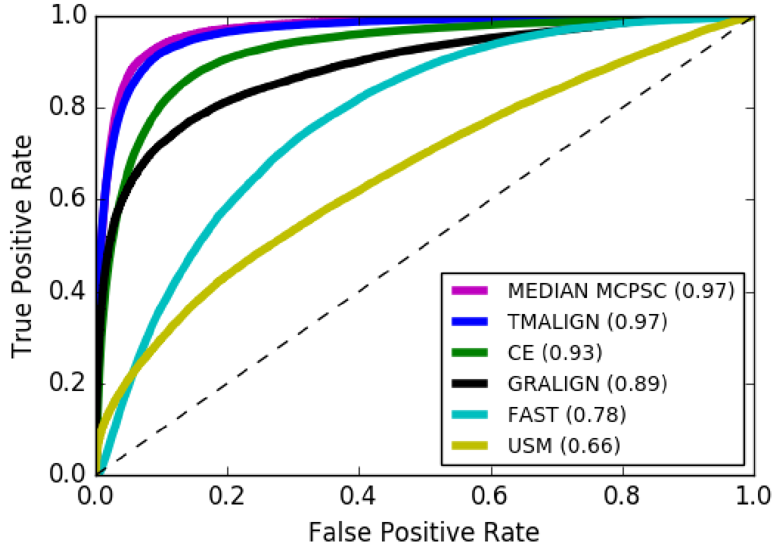

(b) Common Dataset

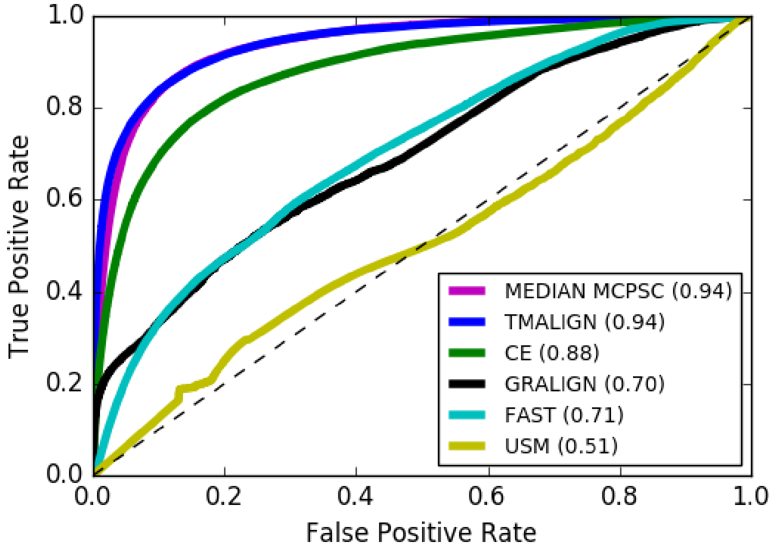

(c) Imputed Dataset

Figure A: ROC curves generated by *pyMCPSC*. The panels are plots for the component PSC methods and median MCPSC method over the three variations of the domain pairs SCOP-CATH dataset. The Area Under the Curve (AUC) is provided in parentheses. The ROCs are generated at the SCOP Superfamily level (Level 3).

	Original dataset				Common subset				Imputed dataset			
**SCOP Level**	1	2	3	4	1	2	3	4	1	2	3	4
TM-align	1.00	0.98	0.91	0.77	1.00	0.74	0.66	0.48	1.00	0.90	0.84	0.71
CE	1.00	0.62	0.56	0.46	1.00	0.49	0.44	0.32	1.00	0.57	0.51	0.42
GRALIGN	1.00	0.91	0.84	0.67	1.00	0.70	0.62	0.47	1.00	0.56	0.47	0.37
FAST	1.00	0.11	0.10	0.09	1.00	0.10	0.09	0.07	1.00	0.11	0.10	0.08
USM	1.00	0.39	0.31	0.24	1.00	0.46	0.38	0.28	1.00	0.36	0.29	0.22
M1	1.00	0.94	0.86	0.68	1.00	0.75	0.66	0.49	1.00	0.85	0.77	0.59
M2	1.00	0.94	0.86	0.68	1.00	0.75	0.66	0.48	1.00	0.86	0.78	0.60
M3	1.00	0.96	0.88	0.70	1.00	0.75	0.66	0.49	1.00	0.87	0.79	0.62
M4	1.00	0.94	0.86	0.68	1.00	0.75	0.66	0.49	1.00	0.85	0.77	0.59
M5	1.00	0.98	0.91	0.76	1.00	0.75	0.67	0.49	1.00	0.90	0.83	0.66
Median MCPSC	1.00	0.94	0.86	0.69	1.00	0.75	0.66	0.49	1.00	0.87	0.79	0.61

Table A: Fraction of domains correctly classified at different SCOP hierarchy levels using a Nearest-Neighbor classifier built with similarity scores produced by different PSC and MCPSC methods. In the SCOP hierarchy: Level 1 = Class, Level 2 = Fold, Level 3 = Superfamily and Level 4 = Family.

B.3 Multidimensional Scaling Scatterplots of protein domains

In Figures B and C we provide the Multi-dimensional Scaling [3] based scatterplot visualizations generated using distance matrices based on the scaled dissimilarity scores of the different PSC methods and MCPSC methods. The figure depicts the domains colored by SCOP class to which they belong. The figures highlight the difference between the spatial arrangements of the different classes and the fairly clear separation between them for the bigger SCOP classes. Domains belonging to Class C (α/β) are split into two major sub-clusters. This points towards an inherent grouping of domains belonging to this class and is potentially a reflection of the presence of two large Fold levels (SCOP level 2) in Class C in this dataset. Analysis of the domains belonging to the two main sub-clusters of Class C revealed that this is in fact the case. One of the sub-clusters has a dominating presence of domains belonging to Architecture level (CATH) *3-Layer (aba) Sandwhich* (64%) while the other is dominated by domains belonging to the Architecture level *2-Layer Sandwhich* (50%). These two Arcitecture levels contribute nearly 1000 domains each to the Class, while the remaining Architectures in the class (13 in all) are significantly smaller. It is also interesting to note from the figure that Class D ($\alpha + \beta$) domains tend to appear at spatial points dominated by points of other Classes. Class D is spread over the entire structural space in general, indicating close structural similarities between members of this Class with those of other SCOP classes.

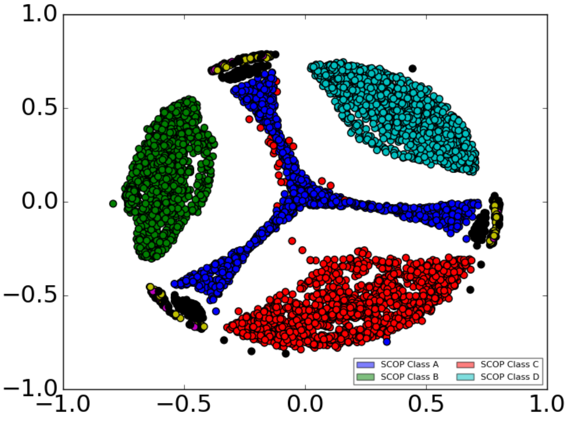

(a) CE

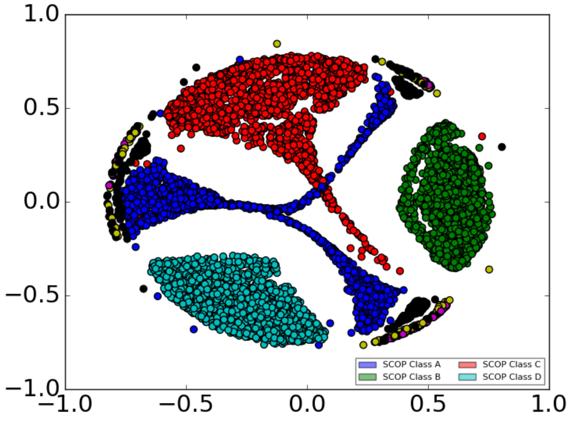

(b) FAST

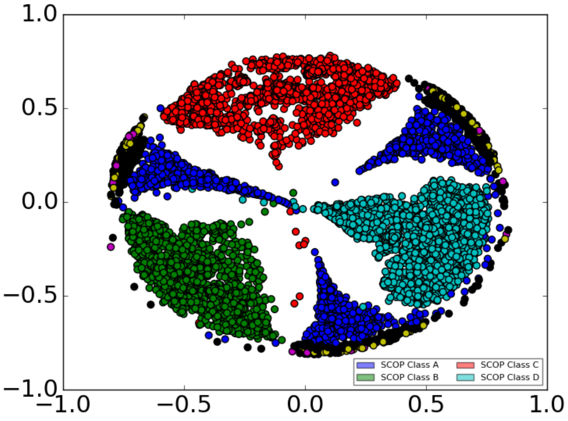

(c) TM-align

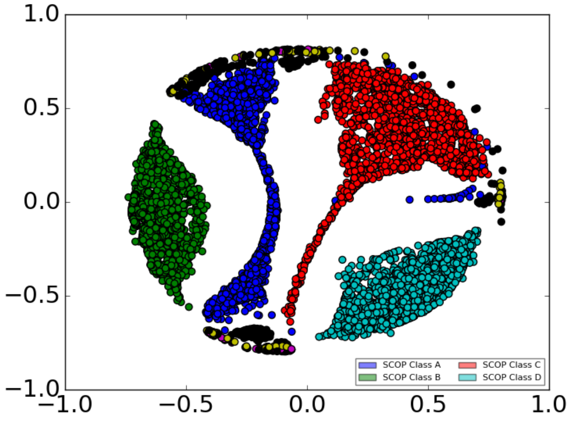

(d) GRALIGN

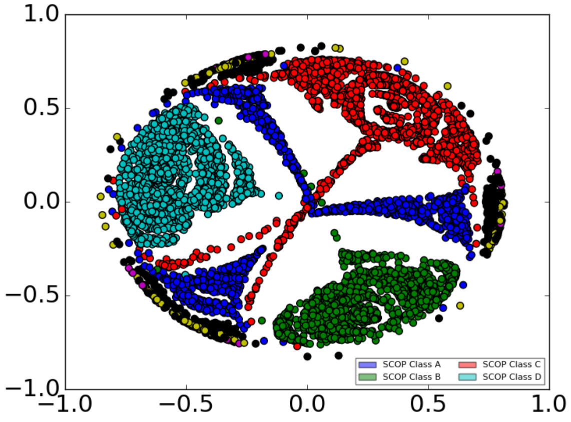

(e) USM

Figure B: MDS scatter-plots for the five PSC methods generated using distance matrices and the imputed dataset. The points are colored by the ground-truth SCOP Level 1 classification of each domain. Blue = SCOP Class A, Green = SCOP Class B, Red = SCOP Class C and Cyan = SCOP Class D. Black points are domains belonging to other classes.

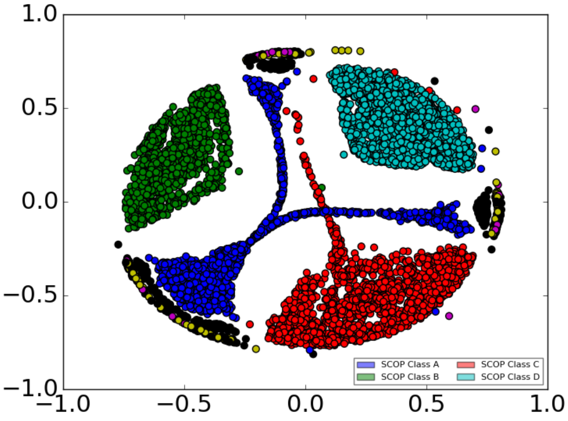

(a) M1

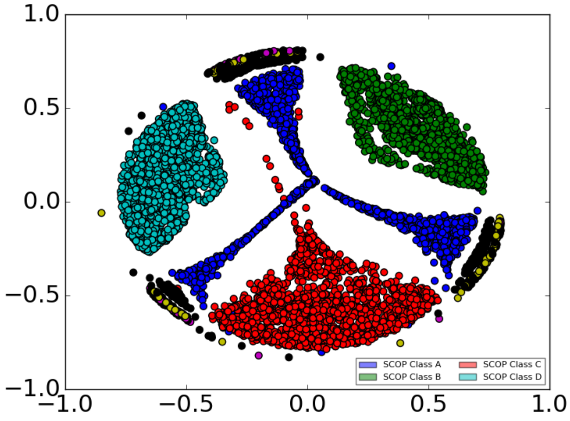

(b) M2

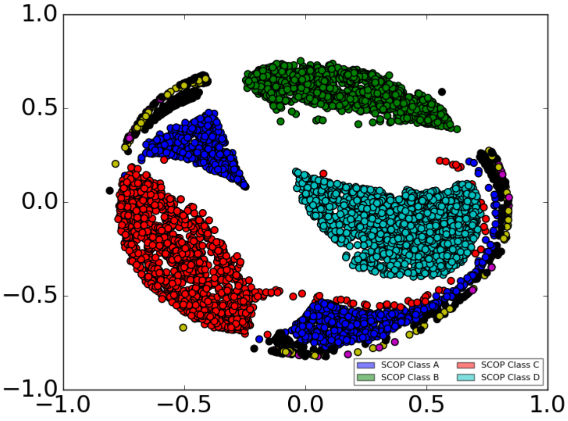

(c) M3

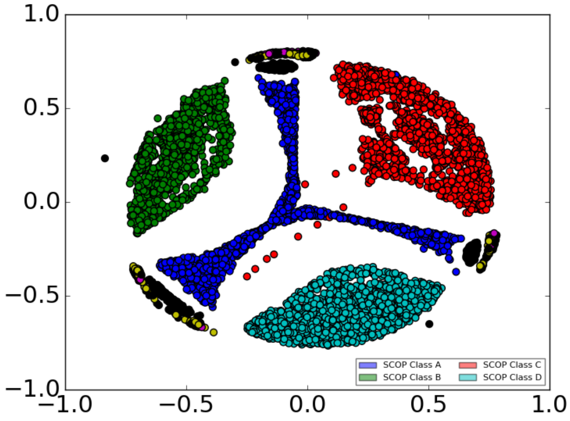

(d) M4

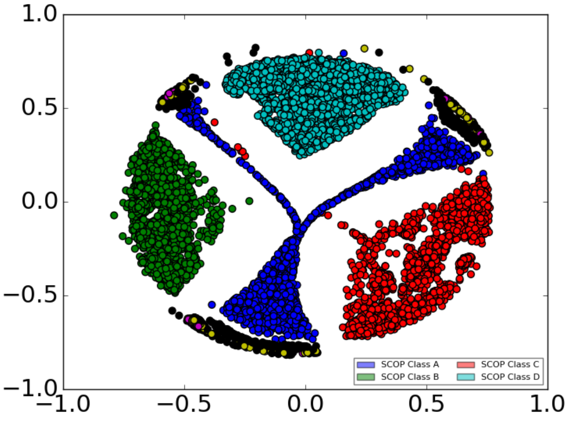

(e) M5

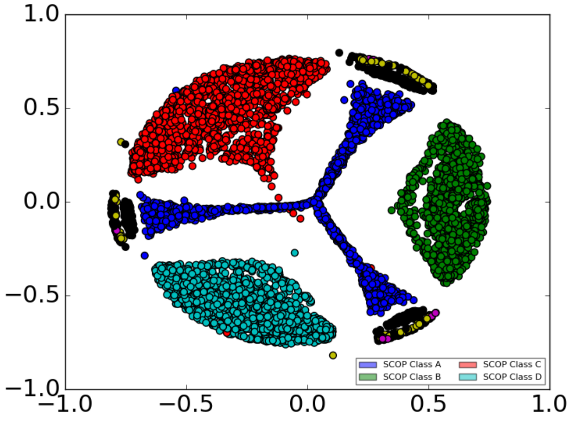

(f) Median

Figure C: MDS scatter-plots for the five MCPSC methods generated using distance matrices and the imputed dataset. The points are colored by the ground-truth SCOP Level 1 classification of each domain. Blue = SCOP Class A, Green = SCOP Class B, Red = SCOP Class C and Cyan = SCOP Class D. Black points are domains belonging to other classes.

References

[1] Gergely Csaba, Fabian Birzele, and Ralf Zimmer. Systematic Comparison of SCOP and CATH: A new Gold Standard for Protein Structure Analysis. *BMC Structural Biology*, 9(23), 2009.

[2] Tom Fawcett. An introduction to roc analysis. *Pattern Recogn. Lett.*, 27(8):861–874, June 2006.

[3] W. S. Torgerson. Multidimensional scaling: I. theory and method. *Psychometrika*, 17:401–419, 1952.
